# Mapping and functional analysis of heterochromatin protein 1 phosphorylation in the malaria parasite *Plasmodium falciparum*

**DOI:** 10.1038/s41598-019-53325-9

**Published:** 2019-11-13

**Authors:** Hai T. N. Bui, Igor Niederwieser, Megan J. Bird, Weiwen Dai, Nicolas M. B. Brancucci, Suzette Moes, Paul Jenoe, Isabelle S. Lucet, Christian Doerig, Till S. Voss

**Affiliations:** 1 Department of Medical Parasitology and Infection Biology, Swiss Tropical and Public Health Institute, 4051 Basel, Switzerland; 2 University of Basel, 4003 Basel, Switzerland; 3 Department of Microbiology, Monash University, Clayton, Victoria, 3800 Australia; 4 The Walter and Eliza Hall Institute of Medical Research, Parkville, Victoria, 3052 Australia; 5 Department of Medical Biology, University of Melbourne, Parkville, Victoria, 3052 Australia; 6 Biozentrum, University of Basel, 4056 Basel, Switzerland; 7 Centre for Chronic Inflammatory and Infectious Diseases, School for Health and Biomedical Sciences, RMIT University, Bundoora, Victoria, 3083 Australia

**Keywords:** Parasite biology, Gene silencing

## Abstract

Previous studies in model eukaryotes have demonstrated that phosphorylation of heterochromatin protein 1 (HP1) is important for dynamically regulating its various functions. However, in the malaria parasite *Plasmodium falciparum* both the function of HP1 phosphorylation and the identity of the protein kinases targeting HP1 are still elusive. In order to functionally analyze phosphorylation of *P. falciparum* HP1 (PfHP1), we first mapped PfHP1 phosphorylation sites by liquid chromatography tandem mass spectrometry (LC-MS/MS) analysis of native PfHP1, which identified motifs from which potential kinases could be predicted; in particular, several phosphorylated residues were embedded in motifs rich in acidic residues, reminiscent of targets for *P. falciparum* casein kinase 2 (PfCK2). Secondly, we tested recombinant PfCK2 and a number of additional protein kinases for their ability to phosphorylate PfHP1 in *in vitro* kinase assays. These experiments validated our prediction that PfHP1 acts as a substrate for PfCK2. Furthermore, LC-MS/MS analysis showed that PfCK2 phosphorylates three clustered serine residues in an acidic motif within the central hinge region of PfHP1. To study the role of PfHP1 phosphorylation in live parasites we used CRISPR/Cas9-mediated genome editing to generate a number of conditional PfHP1 phosphomutants based on the DiCre/LoxP system. Our studies revealed that neither PfCK2-dependent phosphorylation of PfHP1, nor phosphorylation of the hinge domain in general, affect PfHP1′s ability to localize to heterochromatin, and that PfHP1 phosphorylation in this region is dispensable for the proliferation of *P. falciparum* blood stage parasites.

## Introduction

Euchromatin and heterochromatin are the two main structures of chromatin in eukaryotes. While euchromatin is associated with active gene transcription, heterochromatin is associated with heritable gene silencing. Heterochromatin is characterized by the enrichment of heterochromatin protein 1 (HP1) bound to trimethylated histone 3 lysine 9 (H3K9me3)^1–3^. HP1 recruits chromatin modifiers such as H3K9me-specific histone methyltransferases, which in turn methylate H3K9 in neighbouring nucleosomes, thus facilitating the binding of further HP1 proteins and consequently the regional spreading of heterochromatin in a sequence-independent manner^3,4^. In addition to promoting gene silencing and heterochromatin maintenance HP1 also plays roles in centromere function in fission yeast and humans and in DNA replication and repair^5,6^.

HP1 is widely conserved among eukaryotes and consists of three functional domains, namely the N-terminal chromo domain (CD) that binds H3K9me3^1,7,8^, the C-terminal chromoshadow domain (CSD) that mediates HP1 homodimerisation and specific interactions with other regulatory proteins^9–11^, and a variable hinge region located between the CD and CSD domains that has been shown to interact with DNA and/or RNA^12–14^. Some eukaryotes have several HP1 paralogs; for instance, *Schizosaccharomyces pombe* encodes two HP1 variants (Swi6 and Chp2) and mammals possess three HP1 variants (HP1α, HP1β and HP1γ)^3,5^.

The parasitic protist *Plasmodium falciparum*, the causative agent of the most severe form of malaria in humans, possesses a single HP1 ortholog (PfHP1). PfHP1 binds to and co-localizes with H3K9me3 to heterochromatic domains in the subtelomeric regions of all 14 chromosomes and to internal heterochromatic islands on some chromosomes^15–19^. There is no evidence for the presence of either PfHP1 or H3K9me3 in peri-centromeric regions, suggesting that PfHP1 does not contribute to the maintenance of centromere structure and function in this organism^15–18,20^. The subtelomeric and chromosome-internal heterochromatic domains collectively cover over 400 protein-coding genes, most of which belong to *P. falciparum*-specific gene families that encode virulence factors exported to the host erythrocyte^15–18^. In addition, PfHP1 also binds to a small number of euchromatic loci, including the gene encoding the master transcription factor of sexual differentiation PfAP2-G^15,17^. Consistent with a role for PfHP1 in heritable gene silencing, almost all PfHP1-associated genes are expressed in a clonally variant manner^21^. The best-studied example is provided by the *var* gene family that consists of approximately 60 members, each encoding a variant of the erythrocyte membrane protein 1 (PfEMP1) antigen that is exposed on the surface of infected red blood cells (iRBCs)^22–25^. The PfEMP1-dependent binding of iRBCs to endothelial cells and uninfected RBCs leads to parasite sequestration in the microvasculature, which strongly contributes to severe disease^26,27^. Importantly, expression of the *var* gene family is controlled in a mutually exclusive manner (aka singular gene choice), such that at any given time only a single *var* gene is transcribed while all other family members are epigenetically silenced in a PfHP1-dependent manner^28–31^. Switches in *var* gene transcription then lead to clonal antigenic variation of PfEMP1, allowing the parasite to evade adaptive immune responses and establish chronic infection^24,26^.

Using an inducible PfHP1 loss-of-function parasite line, where PfHP1 expression levels can be modulated via the FKBP/DD-Shield-1 conditional expression system^32,33^, we recently identified three important roles for PfHP1 in the biology of blood stage parasites^31^. First, we found that PfHP1 is essential for the heritable silencing of heterochromatic gene families as PfHP1 depletion resulted in the de-repression of almost all *var* genes and many other subtelomeric gene families in the progeny. Second, we demonstrated that PfHP1 depletion leads to a 25-fold increase in sexual conversion rates, with over 50% of parasites in the progeny differentiating into gametocytes (which are required for malaria transmission via the mosquito vector). This striking phenotype was linked to de-repression of the *pfap2-g* locus in absence of PfHP1. Third, we showed that the remaining asexual parasites in the PfHP1-depleted progeny failed to enter S-phase, revealing a crucial role for PfHP1 in the control of proliferation^31^.

Studies in model eukaryotes have shown that HP1 is post-translationally modified, particularly by phosphorylation. Phosphorylation of HP1 regulates various functions in a number of cellular processes in fission yeast and mammals, including heterochromatic gene silencing, mitosis and DNA repair^34,35^. For instance, casein kinase 2 (CK2)-dependent phosphorylation of serine residues in the N-terminal part of Swi6 is important for transcriptional silencing and the recruitment of the histone deacetylase complex SHREC in *S. pombe*^36^. Similarly, in mice the N-terminal phosphorylation of HP1α by CK2 is important for targeting HP1 to heterochromatin as well as for chromosomal stability. While a single substitution of serine 14 with alanine (S14A) impaired the binding of HP1α to H3K9me3 and caused diffuse heterochromatic localization, multiple substitutions of clustered serines (S11A to S14A) hampered chromosomal integrity^37^. In humans, HP1 phosphorylation has been shown to play a role in progression through mitosis. Human HP1α is a substrate of the nuclear Dbf2-related (NDR) kinase; in an NDR-depleted cell line, the lack of HP1α phosphorylation at serine 95 in the hinge domain resulted in chromosome alignment defects, aberrant spindle morphology and a delay in metaphase progression^38^. Furthermore, HP1 phosphorylation has also been shown to play a role the DNA damage response in humans. CK2-dependent phosphorylation of HP1β at threonine 51 was shown to modulate the dispersion of HP1 from chromatin, which in turns facilitates histone H2AX phosphorylation and recruitment of downstream regulators involved in repairing chromosomal DNA breaks^39^.

In contrast to model eukaryotes, the functional role of HP1 phosphorylation in *P. falciparum* and the kinases involved are still unknown. Hence, to begin understanding how PfHP1 function is regulated in *P. falciparum*, we studied PfHP1 phosphorylation using *in vitro* and *in vivo* assays. Liquid chromatography tandem mass spectrometry (LC-MS/MS) analysis of immunoprecipitated native PfHP1 showed that PfHP1 is phosphorylated in its CD and hinge domains. *In vitro* kinase assays revealed that PfHP1 is a substrate of *P. falciparum* CK2 (PfCK2). LC-MS/MS analysis showed that PfCK2 targets three clustered serine residues within the PfHP1 hinge region *in vitro*. By generating conditional PfHP1 phosphomutant cell lines using CRISPR/Cas9-mediated genome editing combined with the DiCre/LoxP system^40,41^, we found that PfCK2-dependent phosphorylation of PfHP1, and phosphorylation of the PfHP1 hinge domain in general, is dispensable for proper PfHP1 localisation, gene silencing, parasite growth and sexual conversion.

## Results

### Identification of phosphorylated PfHP1 residues and parasite kinases phosphorylating PfHP1

**Figure 1:**
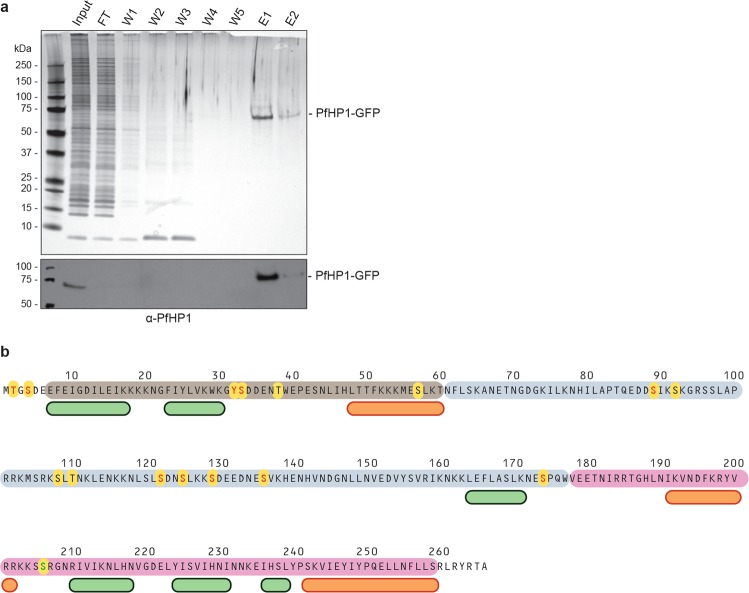
Identification of phosphorylated residues in PfHP1. (**a**) Upper panel: silver-stained SDS-PAGE gel of protein samples from a PfHP1-GFP immunoprecipitation (IP) experiment using nuclear extracts from 3D7/HP1-GFP parasites and GFP-Trap_A beads. Lower panel: Cropped section of a Western blot analysis of PfHP1-GFP IP samples using α-PfHP1 antibodies showing the presence of PfHP1-GFP in the input and elution samples. FT, flow through; W1-W5, wash 1–5; E1/E2, eluates 1 and 2. The full-size blot in shown in Supplementary Fig. 5. (**b**) Overview of all phosphosites (yellow circles) identified in PfHP1, either in this study by LC-MS/MS of immunopurified native PfHP1-GFP or in other proteomics studies^42–48^. Phosphosites identified here and in at least one previous study are highlighted in red letters, those identified only in previous studies are highlighted in black letters, and the phosphosite exclusively identified here (S206) is highlighted by a green letter. Helices and β-sheets predicted by the secondary structure prediction tool JPred4 (http://www.compbio.dundee.ac.uk/jpred4/index.html)^82^ are indicated by orange and green boxes, respectively. The approximate boundaries of the CD (brown), hinge domain (grey) and CSD (pink) are indicated^15^. Numbers refer to amino acid positions in the PfHP1 sequence.

Several large-scale phosphoproteomics studies in *P. falciparum* collectively detected 13 phosphorylated residues in PfHP1 (T2, S4, S33, T38, S57, S89, S92, S108, T110, S122, S125, S129, S174)^42–47^. The Y32 and S136 residues have been identified as additional phosphosites in a recent study investigating native PfHP1 complexes^48^. To confirm and possibly expand these results, we used LC-MS/MS experiments to map phosphorylated residues in native PfHP1. To this end, we purified PfHP1-GFP by immunoprecipitation (IP) from nuclear extracts prepared from 3D7/HP1-GFP schizont stage parasites^31^ in four independent biological replicate experiments (Fig. 1a). LC-MS/MS analysis of the eluted protein samples identified a total of eleven phosphosites in PfHP1 (Fig. 1b and Supplementary Table 1). One of these phosphosites (S206) has not been identified in any of the earlier studies, and five previously mapped phosphosites (T38, S57, S92, S108 and T110) have not been identified here (Fig. 1b). Two of the sites identified in our study are located in the first few residues preceding the CD domain (T2, S4), two are located within the CD domain in a predicted flexible loop (Y32, S33), six are located in the hinge region (S89, S122, S125, S129, S136, S174) and one is located in a predicted loop in the CSD domain (S206) (Fig. 1b).

**Figure 2:**
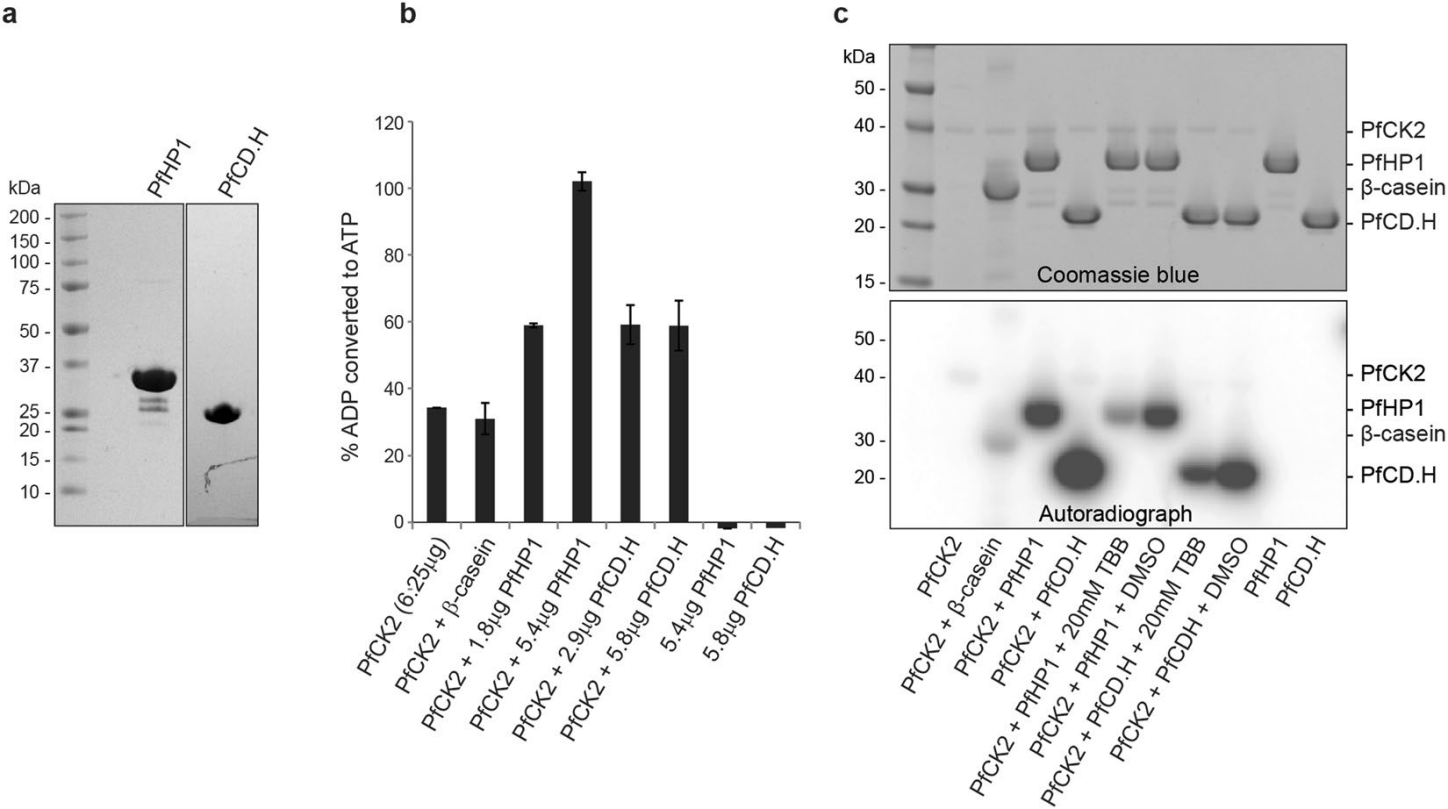
PfCK2 phosphorylates PfHP1 *in vitro*. (**a**) Coomassie-stained SDS-PAGE gel showing the purified recombinant PfHP1 and PfCD.H proteins. (**b**) *In vitro* ADP-Glo assay results reveal that PfCK2 phosphorylates PfHP1 and PfCD.H *in vitro*. The percentage of ADP converted back into ATP (y-axis) is a surrogate measure for kinase activity (i.e. the relative amount of ATP consumed in the kinase reaction). β-casein was included as a positive control substrate for PfCK2. Recombinant PfHP1 and PfCD.H in absence of PfCK2 were used as negative controls. Values represent the average of two replicate reactions. Error bars represent SD. (**c**) Cropped sections of a Coomassie-stained SDS-PAGE gel (top) and the corresponding autoradiogram (bottom) of the *in vitro* γ-P^32^-ATP PfCK2 kinase assay performed with recombinant PfHP1 and PfCD.H substrates. β-casein was used as a positive control substrate. 20 µM TBB was used as a specific inhibitor of PfCK2^50,56^. The full-size Coomassie-stained gel and autoradiogram are shown in Supplementary Fig. 6.

Several of the identified phosphosites conform to CK2 target sites in view of their richness in acidic residues (e.g. S129; SDEE), while others appear as potential targets of proline-directed kinases such as CDK, GSK3 or MAPK (e.g. S174; ESP), or of basic residues-directed kinases such as AGC kinases (e.g. S89; SIK) (Supplementary Table 1) (see Amanchy and colleagues for a comprehensive list of phosphorylation motifs^49^). To identify candidate kinases possibly phosphorylating PfHP1, we screened a set of six recombinant functional parasite kinases, namely PfCK2^50^, PfGSK3^51^, PfMAP2^52^, PfNEK2^53^, PfNEK4^54^ and PfPK6^55^, for their ability to phosphorylate recombinant PfHP1 *in vitro*, which includes the potential candidates PfCK2, PfGSK3 and PfMAP2 predicted by the phosphorylation site motifs outlined above. For this purpose, we used purified recombinant full-length PfHP1 and a truncated PfHP1 polypeptide encompassing the CD domain and hinge region (PfCD.H) (Fig. 2a). To perform the kinase reactions, we used an *in vitro* assay based on luminescence signal detection (ADP-Glo, Promega). Among the recombinant parasite kinases screened, PfMAP2, PfPK6, PfNEK2 and PfNEK4 showed little or no activity on PfHP1 (Supplementary Fig. 1). PfNEK4 phosphorylated the control substrate but not the recombinant PfHP1 proteins. PfMAP2, PfPK6 and PfNEK4 all displayed high autophosphorylation activities as shown previously^52,53,55^ and no increased consumption of ATP was observed in presence of the control substrates or PfHP1, suggesting that these kinases do not target PfHP1; however, we cannot exclude that low-level phosphorylation of PfHP1 may have been masked by the high level of autophosphorylation displayed by these three enzymes. PfGSK3 showed some positive enzymatic activity on PfHP1 but this result was inconclusive due to the high level of autophosphorylation exerted by this kinase both in the ADP-Glo assay as well as in a radioactive kinase activity assay employing γ-P^32^-ATP (Supplementary Fig. 2). Importantly, however, PfCK2 displayed remarkable activity in phosphorylating PfHP1 *in vitro*. In the ADP-Glo assay, PfCK2 showed autophosphorylation activity and was able to phosphorylate the control substrate β-casein. In absence of PfCK2, PfHP1 and PfCD.H exhibited no signals of phosphorylation activity as expected. In contrast, when PfCK2 was added to the PfHP1 and PfCD.H substrates, ADP conversion increased substantially compared to PfCK2 alone or PfCK2 with β-casein (Fig. 2b). Consistent results were obtained for PfCK2 using the *in vitro* γ-P^32^-ATP kinase assay. PfCK2 again showed auto-phosphorylation activity but was clearly capable of phosphorylating β-casein as a positive control and, at substantially higher levels, the PfHP1 and PfCD.H substrates (Fig. 2c). The truncated PfCD.H protein was phosphorylated more efficiently compared to full-length PfHP1, possibly because PfCK2 may have improved access to the target sites in the monomeric recombinant PfCD.H protein compared to the homodimers formed by full-length PfHP1^15^. To further probe the specificity of the CK2-dependent kinase reaction towards PfHP1 and PfCD.H, 4,5,6,7-tetrabromobenzimidazole (TBB), a selective ATP-competitive inhibitor of CK2 across species^56^, was added to the kinase reactions. The treatment with 20 µM TBB resulted in a significant drop of phosphorylation signal intensity in the reactions containing the substrates (Fig. 2c). Finally, to identify the residues in PfHP1 targeted by PfCK2 *in vitro*, we performed LC-MS/MS analysis of the ADP-Glo kinase assay reactions. The results revealed that PfCK2 phosphorylated a cluster of three serine residues in the hinge domain (S122, S125 and S129). Three additional predicted CK2 target residues in the PfHP1 N-terminus (T2, S4, S33; see Supplementary Table 1), however, where not detected in their phosphorylated form. As expected, no phosphorylated sites were detected in recombinant PfHP1 prior to the phosphorylation assay (Supplementary Table 2). We also failed to detect any phosphorylated residues in PfHP1 after incubation with PfGSK3 (Supplementary Table 2).

Overall, using three independent approaches, we demonstrate that PfHP1 is phosphorylated at least at eleven residues during intra-erythrocytic development, that most of these residues are located either at the N-terminus, in predicted loop regions in the CD and CSD domains or in the hinge domain, and that three residues in the hinge region (S122, S125 and S129) are phosphorylated by PfCK2 in *in vitro*.

### Generation of conditional PfHP1 phosphomutant parasite lines

To begin addressing the *in vivo* functional significance of PfHP1 phosphorylation we concentrated on the hinge domain for three reasons. First, the hinge domain contains the majority of phosphorylated residues in PfHP1. Second, we identified PfCK2 as a kinase able to phosphorylate residues in the hinge domain *in vitro*. Third, the poor sequence homology between PfHP1 and HP1 orthologs from model eukaryotes precluded the reliable prediction of functionally important phosphosites in PfHP1.

**Figure 3:**
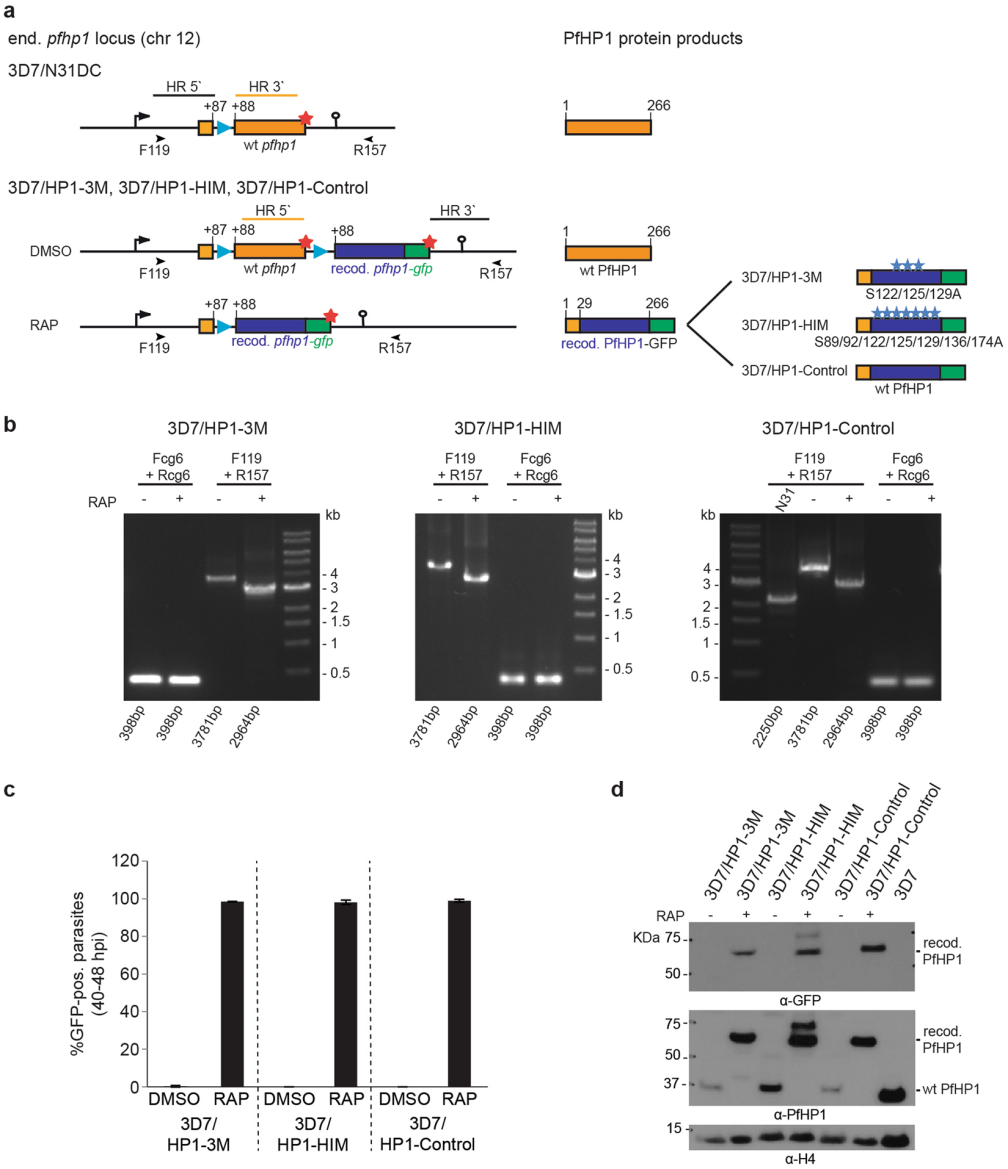
Generation of DiCre-inducible PfHP1 phosphomutants. (**a**) Schematics of the CRISPR/Cas9-edited *pfhp1* loci (left panel) and corresponding PfHP1 protein products (right panel) expressed in the 3D7/N31DC mother line (top) and the 3D7/HP1–3M, 3D7/HP1-HIM and 3D7/HP1-Control parasites (bottom) before (DMSO) and after (RAP) rapamycin-induced DiCre-dependent excision of the wild type *pfhp1* locus. HR, homology regions used for homology-directed repair of Cas9-induced DNA double-strand breaks. Blue arrowheads indicate the position of *sera2* intron:loxP elements^41^. Red asterisks indicate STOP codons. Blue asterisks indicate the relative position of serine-to-alanine substitutions in the PfHP1 hinge region in the 3D7/HP1–3M and 3D7/HP1-HIM phosphomutants. Orange and blue boxes represent the wild type and recodonised *pfhp1*/PfHP1 sequences, respectively. Numbers in the gene and protein schematics refer to nucleotide and amino acid positions, respectively. The black arrowheads indicate the binding sites of the F119 and R157 primers used to confirm correct editing of the *pfhp1* locus and efficient DiCre-mediated excision upon rapamycin treatment by PCR on gDNA (see b below). (**b**) PCR on gDNA confirms the correctly edited *pfhp1* loci and efficient excision of the endogenous *pfhp1* gene after rapamycin treatment in 3D7/HP1–3M, 3D7/HP1-HIM and 3D7/HP1-Control parasites. Using primers F119 and R157 (see a) the correctly edited *pfhp1* locus delivers a PCR product of 3781 bps in the 3D7/HP1–3M (left panel), 3D7/HP1-HIM (middle panel) and 3D7/HP1-Control line (right panel). In the 3D7/N31DC mother line (i.e. prior to the insertion of a recodonised *pfhp1-gfp* fusion gene) this PCR reaction delivers a 2250 bps fragment (right panel). Correct excision of the endogenous *pfhp1* gene in rapamycin-treated 3D7/HP1–3M, 3D7/HP1-HIM and 3D7/HP1-Control parasites results in a decrease of the size of the PCR fragment from 3781 bps to 2964 bps. Primers targeting the *cg6* control locus (PF3D7_0709200) have been used as control. RAP, rapamycin. (**c**) The efficiency of DiCre-mediated excision of the endogenous *pfhp1* gene and resulting expression of the recodonised PfHP1-GFP fusion proteins has been quantified by counting the number of GFP-positive parasites in paired control (DMSO) and rapamycin-treated (RAP) populations in late schizont stages (40–48 hpi) (40 hrs after rapamycin treatment). Values represent the mean of three (3D7/HP1-HIM) and four (3D7/HP1–3M and 3D7/HP1-Control) independent biological replicate experiments (>100 iRBCs scored for each population). Error bars indicate SD. (**d**) Cropped sections of a Western blot showing the expression of endogenous untagged PfHP1 and recodonised PfHP1-GFP in the progeny of DMSO- and rapamycin-treated 3D7/HP1–3M, 3D7/HP1-HIM and 3D7/HP1-Control parasites (16–24 hpi, generation 2) (64 hrs after rapamycin treatment). A sample harvested from 3D7 wild type parasites was used as negative control sample. α-histone 4 (H4) antibodies were used as loading control. RAP, rapamycin. The full-size blots are shown in Supplementary Fig. 7.

To study the potential role of hinge domain phosphorylation in regulating PfHP1 function, we used two subsequent CRISPR-Cas9-based gene editing steps to engineer parasites that allow for the conditional expression of PfHP1 phosphomutants based on the DiCre-loxP system^40^ (Fig. 3a and Supplementary Figs 3 and 4). In the first step, a *sera2* intron:loxP element^41^ was inserted into the 5′ end of the endogenous *pfhp1* gene to obtain the 3D7/N31DC mother line. PCRs on gDNA and cDNA and Sanger sequencing confirmed the correct editing of the *pfhp1* locus and the correct splicing of the *sera2* intron:loxP element (Supplementary Fig. 4). In the second step, a second *sera2* intron:loxP element followed by a recodonised *pfhp1* gene fused to *gfp* was placed directly downstream of the *pfhp1* STOP codon (Fig. 3a and Supplementary Fig. 3). In these parasites, activation of the DiCre recombinase by rapamycin is expected to excise the floxed endogenous *pfhp1* gene and to place a recodonised version encoding a PfHP1-GFP phosphomutant under control of the endogenous promoter (Fig. 3a). We generated two such conditional PfHP1 phosphomutant lines called 3D7/HP1–3M and 3D7/HP1-HIM, where either the three serine residues targeted by PfCK2 *in vitro* (S122/125/129 A) or the cluster of seven phosphorylated serine residues in the hinge region (S89/92/122/125/129/136/174 A), respectively, have been substituted by non-phoshorylatable alanines. We also generated a control cell line where rapamycin treatment results in the replacement of the endogenous *pfhp1* with a recodonized wild type *pfhp1-gfp* sequence (3D7/HP1-Control) (Fig. 3a).

PCR on parasite genomic DNA (gDNA) was used to confirm (1) the correct integration of the recodonised *pfhp1-gfp* gene directly downstream of the endogenous *pfhp1* locus; and (2) the successful DiCre-mediated excision of the floxed endogenous *pfhp1* gene in schizont stages (24–36 hrs after rapamycin treatment) in all three cell lines (Fig. 3b). To confirm correct splicing of the *sera2* intron:loxP element after rapamycin treatment and presence of the mutated codons encoding serine-to-alanine substitutions, RT-PCR and Sanger sequencing was performed (Supplementary Fig. 4). Live cell fluorescence imaging in late schizonts at 40–48 hpi (40 hrs after rapamycin treatment) showed that in each of the three parasite lines, excision of the endogenous *pfhp1* gene was highly efficient and expression of the recodonised PfHP1-GFP variants was observed in close to 100% of parasites in the populations (Fig. 3c). In contrast, parasites in the DMSO-treated control populations did not express GFP-tagged PfHP1 variants as expected. Consistent with the live fluorescence imaging results, analysis of whole parasite protein lysates by Western Blot showed that the rapamycin-treated parasites exclusively expressed the recodonised PfHP1-GFP fusions, while DMSO-treated control parasites exclusively expressed wild-type untagged PfHP1 (Fig. 3d).

### PfHP1 phosphomutants still localize to perinuclear heterochromatin

**Figure 4:**
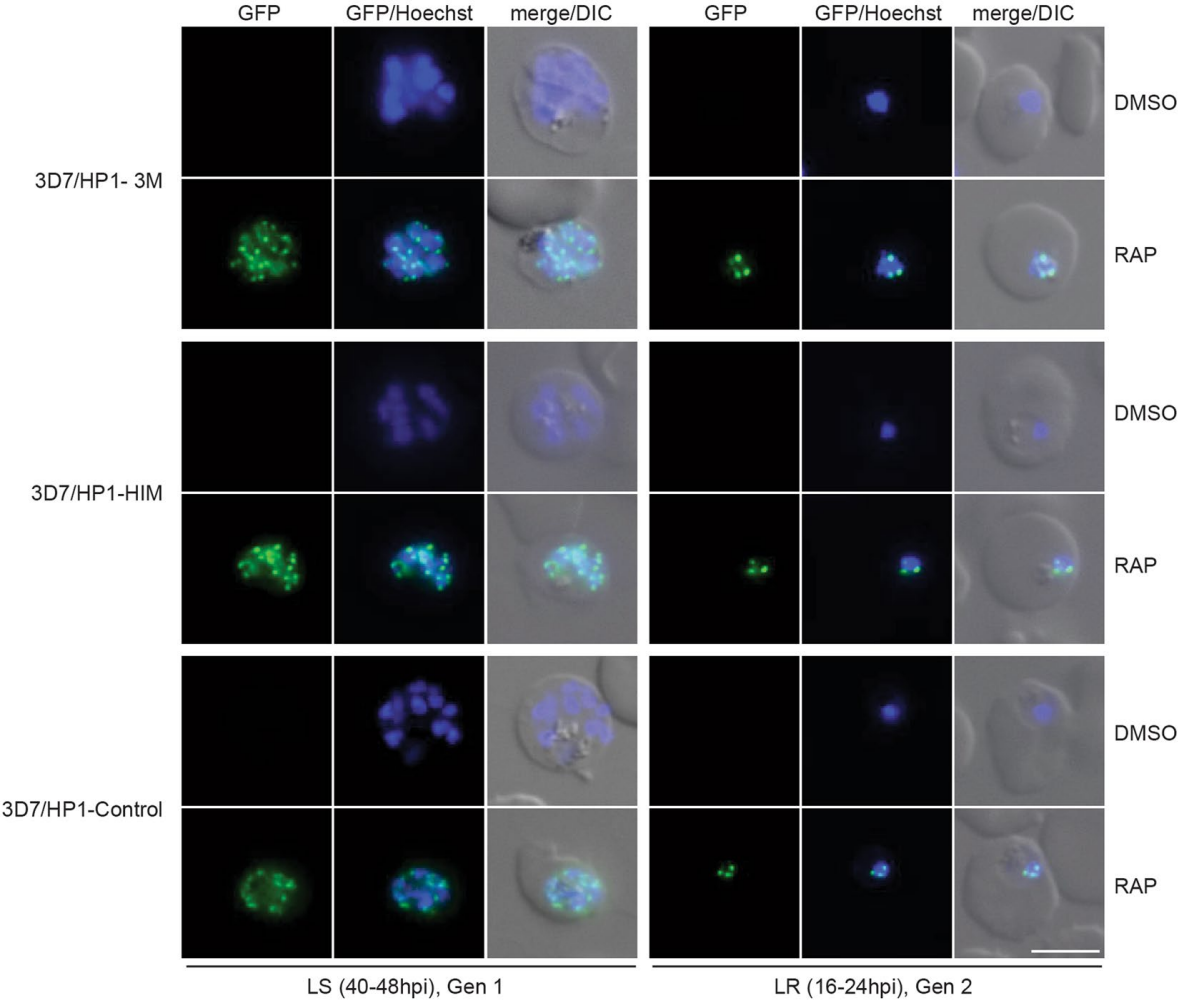
Sub-nuclear localization of PfHP1 phosphomutants. Representative live cell fluorescence images showing the localization of the GFP-tagged PfHP1–3M and PfHP1-HIM phosphomutants and the PfHP1-Control protein in late schizonts (LS, 40–48 hpi, generation 1; 40 hrs after rapamycin treatment) and after re-invasion in the progeny at late ring stage (LR, 16–24 hpi, generation 2; 64 hrs after rapamycin treatment). Nuclei were stained with Hoechst. DIC, differential interference contrast. Scale bar, 5 µm.

In *Drosophila melanogaster* and mice, CK2-dependent phosphorylation of HP1 is required for the correct localization of HP1 to heterochromatin^37,57^. We therefore tested if the PfHP1 phosphomutants PfHP1–3M and PfHP1-HIM still localize to subtelomeric heterochromatin. Live cell fluorescence imaging in late schizonts at 40–48 hpi and in the late ring stage progeny at 16–24 hpi in generation 2 showed that the GFP-tagged PfHP1–3M, PfHP1-HIM and control PfHP1-GFP were not expressed in DMSO-treated parasites as expected. However, in the rapamycin-treated populations the GFP-tagged PfHP1–3M and PfHP1-HIM phosphomutants were expressed and showed a punctate pattern at the nuclear periphery indistinguishable from that observed for the PfHP1-GFP control protein (Fig. 4). These results demonstrate that the phosphorylation of serine residues in the PfHP1 hinge domain is not required for the correct targeting and localization of PfHP1 to heterochromatin.

### Phosphorylation of serine residues in the PfHP1 hinge domain is not required for parasite multiplication and plays no obvious role in regulating gene silencing

**Figure 5:**
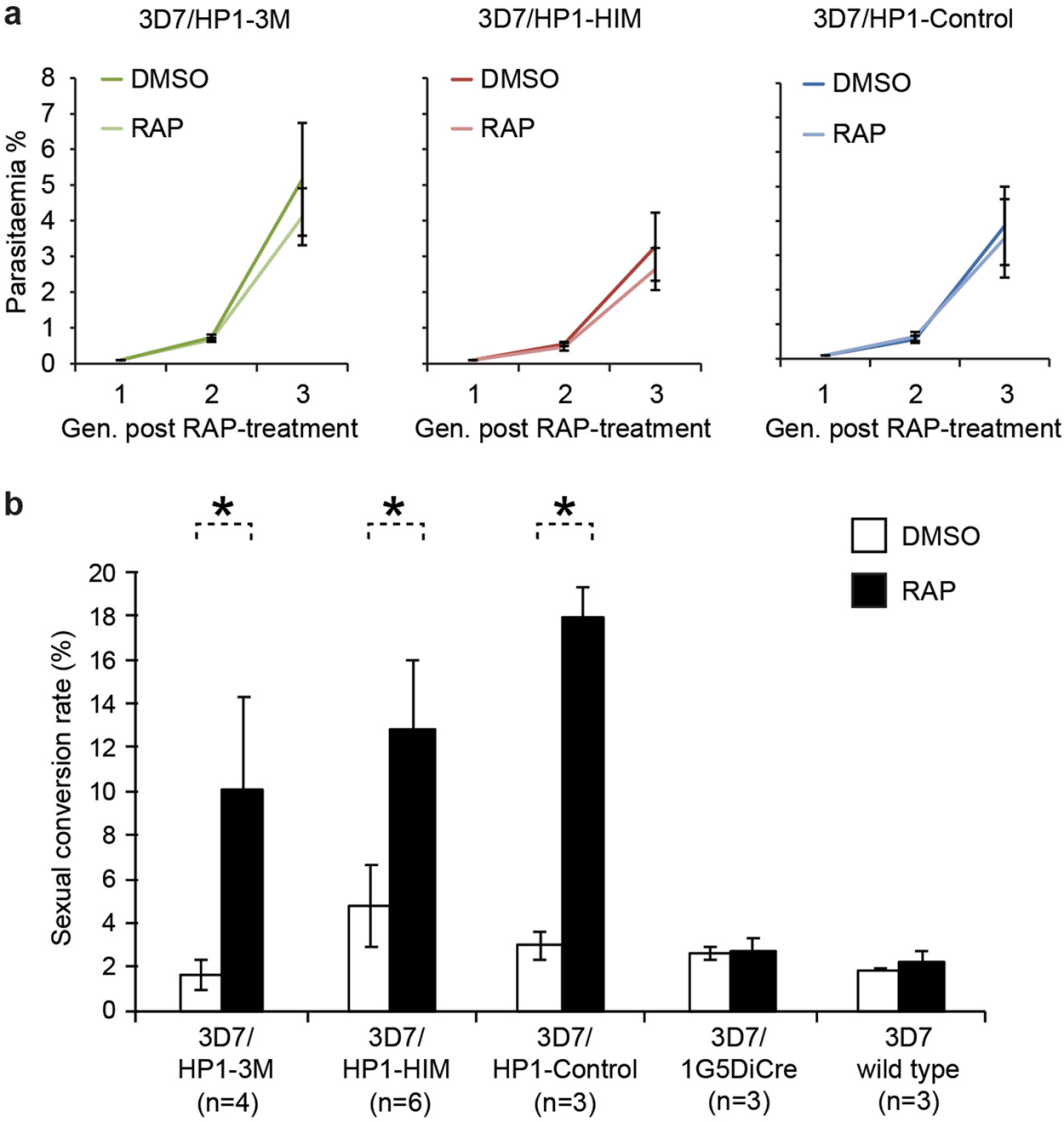
Phenotypes of PfHP1 phosphomutants. (**a**) Growth curves of the control (DMSO) and rapamycin-treated (RAP) PfHP1-GFP phosphomutants and control line over three generations of intra-erythrocytic replication. Values are the mean of four (3D7/HP1–3M and 3D7/HP1-Control) and six (3D7/HP1-HIM) independent replicate experiments. Error bars represent SD. (**b**) Sexual conversion rates of DMSO- and rapamycin-treated (RAP) PfHP1-GFP phosphomutants, the PfHP1-Control line, the 3D7/1G5DiCre mother line and 3D7 wild type parasites. Values represent the mean of three to six independent replicate experiments. Error bars represent SD. Asterisks indicate significant differences in sexual conversion rates between RAP-treated and DMSO-treated parasites (p < 0.01; unpaired two-tailed Student’s t-test).

In a recent study, we showed that PfHP1 is required for parasite multiplication and for heritable gene silencing^31^. Here, we asked if phosphorylation of serine residues in the PfHP1 hinge domain is required for any of these crucial functions. We first monitored the proliferation rates of the PfHP1 phosphomutants and the control line over three consecutive generations after rapamycin treatment. As shown in Fig. 5a, in each parasite line the multiplication of DMSO- and rapamycin-treated parasites was highly comparable. This result indicates that phosphorylation of the PfHP1 hinge domain is not required for the proliferation of asexual blood stage parasites.

To identify a possible role for PfHP1 hinge domain phosphorylation in regulating *pfap2-g* silencing, we compared the sexual conversion rates between DMSO- and rapamycin-treated parasites for all three transgenic parasite lines. Parasite populations were split at 0–8 hpi and treated either with DMSO or rapamycin. After re-invasion, ring stage progeny (16–24 hpi in generation 2; day 1 of gametocytogenesis) were cultured in medium containing 50 mM N-acetyl-glucosamine (GlcNAc) for six consecutive days to eliminate asexual parasites^58,59^. Gametocytaemia was determined on day 6 by inspection of Giemsa-stained blood smears and sexual conversion rates were calculated as the fraction of gametocytaemia on day 6 in relation to the total parasitaemia observed on day 1. As shown in Fig. 5b, the rapamycin-treated 3D7/HP1–3M and 3D7/HP1-HIM populations showed significantly higher sexual conversion rates compared to the DMSO-treated populations (p < 0.01, unpaired two-tailed Student’s t-test). Unexpectedly, rapamycin-treated 3D7/HP1-Control parasites, which express a recodonised wild-type *pfhp1* gene, also displayed significantly increased sexual conversion compared to the matched DMSO-treated population. In contrast, however, the 3D7/1G5DiCre mother line, which expresses the DiCre recombinase in absence of a floxed *pfhp1* locus, as well as 3D7 wild type parasites did not show increased sexual conversion rates upon rapamycin treatment, showing that neither the rapamycin-induced expression of the DiCre recombinase *per se* nor the exposure to rapamycin affected the sexual conversion rates. Hence, the increased sexual conversion rates observed for rapamycin-treated 3D7/HP1–3M, 3D7/HP1-HIM and the 3D7/HP1-Control parasites rather seem to be linked to the DiCre-dependent recombination events at the floxed *pfhp1* locus. Whatever the mechanisms underlying this puzzling observation, our data clearly suggest that phosphorylation of the hinge domain plays no important role in regulating *pfap2-g* silencing. Consistent with this result, preliminary experiments based on indirect immunofluorescence experiments showed similar PfEMP1 expression levels in DMSO- and rapamycin-treated parasites of all three lines, suggesting that hinge domain phosphorylation plays also no major role in PfHP1-dependent silencing of subtelomeric virulence genes.

## Discussion

Heterochromatin-dependent gene silencing is an important mechanism employed by *P. falciparum* for clonally variant gene expression. PfHP1 is one of the critical factors in this process. In addition, PfHP1 is essential for proliferation of blood stage parasites. Phosphorylation of HP1 has been reported to dynamically regulate the function of this chromatin reader protein in a variety of eukaryotic organisms. Our study described here analysed phosphorylation of PfHP1 and its potential functional role in the biology of *P. falciparum* blood stage parasites.

Using immunoprecipitation of native PfHP1-GFP followed by LC-MS/MS analysis we identified eleven phosphorylated residues in PfHP1 purified from schizont stage parasites. All except one of these sites (S206) have either been identified on multiple tryptic peptides and/or in at least two of the independent IP samples analysed here, or they have been detected in previous studies by high-throughput phosphoproteomics approaches^42–47^ and/or the mass spectrometry-based analysis of PfHP1 complexes^48^. Hence, we assume these residues are truly phosphorylated in intra-erythrocytic parasites. Five additional PfHP1 phosphosites (T38, S57, S92, S108, T110) that were identified in previous phosphoproteomics studies were not detected in our immunoprecipitated PfHP1 samples. This may be explained by the fact that in contrast to these previous studies we didn’t enrich for phosphopeptides prior to LC-MS/MS analysis. In such raw tryptic digests the detection of phosphopeptides is often suppressed by the presence of the corresponding non-phosphorylated peptides. Furthermore, phosphosite detection by mass spectrometry is not fully comprehensive and indeed none of the previous phosphoproteomics studies detected all PfHP1 phosphosites simultaneously. Alternatively, it is also likely that differences in parasite stage composition between the samples analysed in the various studies may account for differential phosphosite detection.

By screening a number of recombinant *P. falciparum* kinases using two independent kinase activity assays, we found that PfCK2 and PfGSK3 are able to phosphorylate PfHP1 *in vitro*. However, for PfGSK3 the phosphorylation signals obtained with the control and PfHP1 substrates were not noticeably higher compared to those obtained from auto-phosphorylation. GSK3 is a serine/threonine protein kinase preferring to catalyse substrates upon prior phosphorylation of a residue nearby the consensus sequence (S*/T*-X-X-X-S/T)^60,61^. Therefore, it is likely that GSK3 is only able to efficiently phosphorylate substrates if they have been primed by phosphorylation through another kinase, which was not applied in our *in vitro* kinase assays. Moreover, although PfGSK3 phosphorylated PfHP1 in the ADP-Glo assay and to some extent also in the radioactive kinase assays, no PfGSK3-dependent phosphosites were detected on the *in vitro*-reacted PfHP1 substrates by LC-MS/MS analysis. Hence, additional experiments with higher sensitivity will be required to confirm if PfHP1 is indeed phosphorylated by PfGSK3.

Importantly, however, we obtained convincing results showing that PfCK2 phosphorylates PfHP1 at a cluster of three serine residues located in the hinge region (LSLS122*DNS125*LKKS129*DEE), at least *in vitro*. Serine 122 and S129 are embedded in a typical CK2 phosphorylation consensus target motif rich in acidic residues (S*/T*-D/E-X-D/E)^62–64^, whereas S125 lacks the favoured acidic residue at position +1 or +3 as the most crucial specificity determinant of the phosphoacceptor site^65^. However, similar atypical CK2 recognition sites containing only one acidic residue between −1 to +5 have indeed been reported^66^. In our efforts to analyse the role of PfCK2-dependent PfHP1 phosphorylation *in vivo*, however, we found that substituting the three target residues in the hinge domain with non-phosphorylatable alanines (S122/125/129A; PfHP1–3M) had no observable effect on PfHP1 function; parasites expressing the PfHP1–3M triple phosphomutant displayed no marked defects in PfHP1 localisation, *pfap2-g* silencing or mitotic progression. Strikingly, we even failed to observe any silencing- or growth-related phenotypes in parasites expressing the PfHP1-HIM phosphomutant, where seven serine residues in the hinge region have been replaced with alanines (S89/92/122/125/129/136/174A).

In line with these results, it has been reported that phosphorylation of S93 within the mouse HP1α hinge region or S89/91 within the human HP1β hinge domain does not affect HP1’s localization to chromatin^37,39^. In addition, co-immunoprecipitation of human HP1β with histone 3 revealed only a minor role for S89 phosphorylation in the ability of HP1β to bind H3K9me3, in spite of local conformational changes induced upon phosphorylation^67^. In another study on Swi6, the HP1 ortholog in *S. pombe*, mutations of CK2-dependent phosphosites in the hinge and CSD domains (S192/212/220/268/274 A) did also not affect heterochromatic gene silencing^36^. In other systems, however, essential roles of phosphorylation within the HP1 hinge region have still been reported. In humans, the Aurora A- and NDR1-dependent phosphorylation of serine residues in the hinge region of HP1γ and HP1α, respectively, both play crucial roles in mitotic progression during the G2/M phase^68,69^. Further, protein kinase A (PKA)-dependent phosphorylation of the hinge domain of a *D. melanogaster* HP1 variant plays important roles in HP1 dimerisation, protein-protein interaction and binding to H3K9me3^70^.

In conclusion, our study confirms that PfHP1 is phosphorylated during intra-erythrocytic development at multiple residues particularly in the CD and hinge domains. We also show that PfCK2 phosphorylates three clustered serine residues in the PfHP1 hinge region *in vitro*, but the phosphorylation of these sites is not required for proper PfHP1 function in blood stage parasites. If S122, S125, and S129 are indeed phosphorylated by PfCK2 *in vivo* and if PfCK2 phosphorylates additional target residues in PfHP1 could be tested in future experiments employing conditional PfCK2 knock-down cell lines^71^. Surprisingly, we also demonstrate that the simultaneous mutation of seven phosphorylation target residues in the hinge domain has no observable effect on parasite viability. We therefore anticipate that phosphorylation of the PfHP1 hinge region may play an important role elsewhere in the parasite life cycle, for instance during meiosis in the mosquito vector and/or in the replicative phases during male gametogenesis, sporogony or exo-erythrocytic schizogony. While our study does not provide functional insight into the role of PfHP1 hinge domain phosphorylation, the experimental pipeline established in this study provides an elegant approach to interrogate protein function and the role of post-translational protein modifications in the biology of *P. falciparum* blood stage parasites.

## Methods

### Parasite culture and transfection

3D7/HP1-GFP parasites^31^ were cultured at 5% hematocrit in RPMI-1640 medium supplemented with 25 mM HEPES, 100 mM hypoxanthine, 24 mM sodium bicarbonate and 0.5% Albumax II. The transgenic lines generated in this study were cultured in the same medium supplemented with 2 mM choline to reduce background sexual conversion rates as demonstrated recently^72^. Parasite cultures were synchronized using 5% sorbitol as described^73^. Co-transfection of CRISPR/Cas9 and donor plasmids into the DiCre-expressing line 3D7/1G5DiCre^40^ and selection of transfected populations was performed as described recently^48^.

### Immunoprecipitation of native PfHP1-GFP

Parasite nuclei were isolated from 30 ml culture of 3D7/HP1-GFP early-to-late schizont stage parasites (5% hematocrit) as described previously^48^. Nuclear proteins were extracted using extraction buffer (2 M L-arginine, 1.925 M HCl, 50 mM H_3_PO_4_, and 10 mM TCEP) for 20 min on ice. The extract was cleared by centrifugation for 20 min at 20’000 g and 4 °C. The supernatant was diluted 1:5 with wash buffer 1 (WB1) (PBS containing additional 324 mM NaCl, 1 M L-proline, 1% octyl β-D-glucopyranoside and 2 mM TCEP) and spun again using the same conditions. Extraction buffer, WB1 and buffers used for nuclear isolation were supplemented with 1x protease inhibitor cocktail (Roche), 5 mM ε-aminocaproic acid (protease inhibitor), 3 mM sodium butyrate (histone deacetylase inhibitor) and 2 mM NaF, 2 mM β-glycerophosphate, 4 mM sodium tartrate, 1 mM sodium pyrophosphate and 1 mM activated NaVO_3_ (phosphatase inhibitors). GFP-Trap_A beads (Chromotek) were equilibrated in WB1, added to the supernatant and rotated for 1 h at room temperature (RT). The beads were washed three times with WB1 and twice using WB2 (PBS containing additional 824 mM NaCl and 0.2 mM TCEP). Proteins were eluted using arginine elution buffer (2 M L-arginine, 50 mM acetic acid and HCl to pH 4). The eluate was neutralized by addition of 0.1 volumes of 1 M tris base. Next, the samples were processed and analyzed using LC-MS/MS as detailed below.

### Capillary liquid chromatography-tandem mass spectrometry (LC-MS/MS)

For the PfHP1-GFP IP experiments, the neutralised elutions were reduced with 10 mM DTT at 37 °C for 1 hr and alkylated with 50 mM iodo-acetamide for 15 min at  RT. Proteins were digested with 250 ng endoproteinase LysC (Wako, Neuss, Germany) for two hours at 37 °C followed by 500 ng trypsin (Worthington, Lakewood, NJ, USA) overnight. The digest was stopped with TFA to 1% final concentration and desalted on a microspin column (The Nest Group, Southborough, MA, USA) according to the manufacturer’s recommendations.

For the ADP-Glo *in vitro* kinase assay samples, 20 µl of the technical duplicate reactions were pooled, precipitated with 20% trichloroacetic acid (TCA) on ice for 30 min, followed by washing the precipitate with 17% TCA and two acetone (ice-cold) washes before air-drying the pellets. The protein pellets were dissolved in 30 μl 100 mM Tris-HCl (pH 8.0)/6 M Urea, reduced and alkylated as above and digested with 250 ng endoproteinase LysC for two hours at 37 °C. The urea concentration was diluted to 2 M with 100 mM Tris-HCl (pH 8.0) and the sample was further digested with 500 ng trypsin overnight at 37 °C. The digest was acidified with 1% TFA and the sample was desalted on a MicroSpin cartridge according to the manufacturer’s recommendations.

The eluted peptides were dried in a SpeedVac and dissolved in 40 μL 0.1% formic acid and analysed by capillary LC-MS/MS using a home-packed separating column (0.075 mm × 25 cm) packed with Reprosil C18 reverse-phase material (2.4 μm particle size, Dr. Maisch, Ammerbuch-Entringen, Germany). The column was connected on line to an Orbitrap Elite FT hybrid instrument (Thermo Scientific, Reinach, Switzerland). The solvents used for peptide separation were 0.1% formic acid in water (solvent A) and 0.1% formic acid and 80% acetonitrile in water (solvent B). 2 μl of peptide digest were injected with a Proxeon nLC capillary pump (Thermo Scientific) set to 0.3 μl/min. A linear gradient from 0 to 40% solvent B in solvent A in 95 min was delivered with the nano pump at a flow rate of 300 nl/min. After 95 min the percentage of solvent B was increased to 75% in ten minutes. The eluting peptides were ionized at 2.5 kV. The mass spectrometer was operated in data-dependent mode. The precursor scan was done in the Orbitrap set to 60,000 resolution, while the fragment ions were mass analyzed in the LTQ instrument. A top twenty method was run so that the twenty most intense precursors were selected for fragmentation. The MS/MS spectra of the four PfHP1-GFP IP samples were searched against a combined *P. falciparum* (www.plasmoDB.org; release 9.3)/human annotated protein database using Proteome Discoverer 2.2 (Thermo Scientific, Reinach, Switzerland) using the two search engines Mascot and SequestHT (Supplementary Table 1). The PfHP1-GFP IP replicate samples 1 and 2 were additionally searched against the PfHP1-GFP sequence (Supplementary Table 1). The PfCK2 and PfGSK3 ADP-Glo *in vitro* kinase assay samples were searched against the respective PfHP1 and PfCD.H recombinant protein sequences (Supplementary Table 2). For the search, oxidized methionine, N-terminal protein acetylation and phosphorylation on serine, threonine and tyrosine were used as variable modifications. The identifications were filtered for a false discovery rate of 1%.

### Generation of *E. coli* expression vectors

In order to increase the solubility strength of the SUMO tag, sequences encoding additional solubility tags were inserted upstream of the sequence encoding an N-terminal 6xHis-SUMO tag as suggested elsewhere^74^. For this purpose, the His-SUMO-encoding sequence was PCR amplified from pETA-HS^17^ using the primers Bsa_His_f and T7term. The *Bsa*I/*Xho*I-digested product was then cloned into *BamH*I/*Xho*I-cut pGB1 (a kind gift of S. Hiller) and pETA-MBP^75^, yielding the GB1-His-SUMO (pETA-GHS) and MBP-His-SUMO (pETA-MHS) expression vectors, respectively. Two gene fragments were amplified from 3D7 genomic DNA; full-length *pfhp1* (using primers HP1_F and HP1_Xho_R) and a truncated version encoding the CD and hinge domains only (PfCD.H: M1-T181 (using primers HP1_F and CDH_Xho_R). *Xho*I-digested PCR products were ligated into *Sfo*I/*Xho*I-cut expression vectors; full-length *pfhp1* was cloned into pETA-MHS and *pfcd.h* into pETA-GHS. All primer sequences are listed in Supplementary Table 3.

### Expression and purification of recombinant PfHP1

Both recombinant proteins were expressed in *E. coli* Rosetta2 (DE3) cells (Novagen) using auto-induction at 22 °C in ZYM-5052 medium^76^. Expression cultures were spun down at 4 °C and the pellets were kept at −20 °C. Both recombinant proteins were purified using nickel affinity (A), followed by dextrin affinity (MHS-HP1 only) (B) and tag removal (C). PfCD.H was further purified using hydrophobic interaction chromatography (HIC) (D). Both proteins were polished using gel filtration (E). All affinity columns used were produced by GE Healthcare. (A) Nickel affinity. *E. coli* pellets were resuspended in buffer N-A (50 mM H_3_PO_4_, 20 mM imidazole, 500 mM NaCl, 5 mM EACA and NaOH to pH 7.4) and lysed by sonication. The lysates were loaded on 1 ml HisTrap columns, washed with 20 column volumes (CV) of the same buffer and eluted using N-B (50 mM H_3_PO_4_, 225 mM imidazole, 500 mM NaCl, 5 mM EACA). For full length PfHP1, 2 M urea was included in the lysis buffer. The nickel eluate containing PfCD.H was buffer exchanged to subtraction buffer (0.75x concentrated N-A complemented with 10% glycerol, 1 mM TCEP and additional 125 mM NaCl) using three 5 ml HiTrap desalting columns. (B) Dextrin affinity (MHS-HP1 only). The protein was eluted from the nickel column directly on a 5 ml MBPTrap HP column placed below the HisTrap column. After elution, the nickel column was removed and the MBPTrap column was washed with 5 CV of N-A and eluted with N-A containing 2 M urea, 10 mM maltose and 1 mM TCEP. (C) Tag removal. The GHS and MHS tags were cleaved off using recombinantly expressed SUMO protease (L403-K621 of *S. cerevisiae* ULP1; expressed as GB1-ULP1–6xHis fusion and purified by nickel affinity and gel filtration) in a ratio of 1:200 and incubated for 1.5 hours at 16 °C. In order to subtract the tag, the protease and other contaminants, the digest was passed through a HisTrap column. In the case of full-length PfHP1, guanine-HCl was added to a final concentration of 1 M for this step. (D) HIC (PfCD.H only). Ammonium sulfate from a 4 M stock (pH adjusted to 7 using NH_4_OH) was added to the protein sample to 1.5 M and this mixture was loaded on a 1 ml Phenyl HP column equilibrated in buffer HIC (1.5 M ammonium sulfate, 5 mM EACA, 0.5 mM EDTA, 20 mM H_3_PO_4_-KOH, pH 6.8, and 10% Glycerol). The column was washed with 20 CV of the same buffer and the protein eluted using a 25 CV long linear gradient, from 1.5 M to 0 M ammonium sulfate. (E) Gel filtration. PfCD.H was polished using 10 mM MOPS-KOH, pH 7, 100 mM NaCl, 10% glycerol buffer and a Superdex75 10/300 GL column. For full-length PfHP1, a HiLoad 26/60 Superdex 200 column was prepared in three steps: first, it was equilibrated in storage buffer (20 mM MOPS-KOH, pH 7, 0.5 M NaCl, 10% glycerol). Then, a gradient (1/10th CV) from storage buffer to refolding buffer (20 mM MOPS-KOH, pH 7, 800 mM arginine, 267 mM citric acid) was loaded to the column, and finally a second gradient (1/8th CV), from refolding buffer to buffer N-A containing 2 M urea and 1 M guanine-HCl was loaded. As a result, the protein passed first through refolding and later through storage buffer. Both proteins were concentrated using Amicon spin filter (Millipore) with a 10 K cut-off and stored at −80 °C. Their purity and concentration were determined by SDS-PAGE and NanoDrop™ 2000/2000c spectrophotometer with UV extinction coefficients calculated by protparam (https://web.expasy.org/protparam/).

### *In vitro* kinase assays

Purified recombinant *P. falciparum* kinases were prepared as described for PfCK2^50^, PfGSK3^51^ and PfMAP2, PfNEK2, PfNEK4, PfPK6^77^. The ADP-Glo *in vitro* kinase assay was performed in duplicates according to the manufacturer’s instructions (Promega, USA). In this assay, the enzymatic reaction starts when a kinase is added into a mixture containing the substrate and ATP. Upon completion of the reaction, the ADP-Glo assay quantifies the levels of ADP released from consumed ATP as a measure of kinase activity. The exact amount of recombinant kinase (0.6–7 μg) and PfHP1 or PfCD.H (1.8–5.8 μg) substrates used in each reaction is indicated in the corresponding figures. 10 µg of either histone from calf thymus, bovine myelin basic protein (MBP) or β-casein from bovine milk (Sigma-Aldrich) were used as positive controls. 5 µl out of 25 µl of the kinase reactions were used for the final ATP depletion and detection steps. The remaining reaction volume was used for LC-MS/MS analysis to identify phosphosites (see above).

The γ-P^32^-ATP *in vitro* kinase assays were performed in a standard 25 µl reaction in kinase buffer (20 mM Tris HCl pH 7.5, 20 mM MgCl_2_, 2 mM MnCl_2_, 10 mM glycerolphosphate and 10 mM NaF) containing 10 µM ATP, 5 µCi γ-P^32^-ATP (3000 Ci/mmol, Amersham Biosciences), substrates (5.4 µg PfHP1, 5.8 µg PfCD.H, 10 µg β-casein, 10 µg calf thymus histone, or 5 µg MBP) and recombinant kinases (0.5 µg PfCK2 or 0.6 µg PfGSK3). In the assay using PfCK2, 20 µM of 4,5,6,7-tetrabromobenzimidazole (TBB) in DMSO was use as a specific CK2 inhibitor^50,56^. The reactions were carried out for 30 min at 30 °C and stopped by the addition of Laemmli buffer. The samples were analyzed by SDS-polyacrylamide gel electrophoresis followed by autoradiography.

### Transfection constructs

We applied CRISPR/Cas9-mediated genome editing and the DiCre/LoxP system^40,41^ to generate parasite lines for the conditional expression of PfHP1-GFP phosphomutants. We engineered (1) 3D7/N31DC_PfHP1–3M (3D7/HP1–3M) for expression of the S122/125/129A PfHP1 mutant; (2) 3D7/N31DC_PfHP1-HIM (3D7/HP1-HIM) for expression of the S89/92/122/125/129/136/174A PfHP1 mutant; and (3) 3D7/N31DC_PfHP1-Control (3D7/HP1-Control) for expression of wild type PfHP1-GFP. To obtain these cell lines we performed two subsequent transfection steps.

In the primary transfection, we generated the mother cell line 3D7-1G5DC/5′-loxPint-g31 (3D7/N31DC), which carries a *sera2* intron:loxP element^41^ inserted into the 5′ end of the *pfhp1* coding sequence. To achieve this, we constructed the pHF-gC-guide31 plasmid by inserting two annealed complementary oligonucleotides (F-g31 and R-g31) encoding the sgRNA target sequence and containing appropriate single-stranded overhangs into the *Bsa*I-digested pHF-gC SpCas9 plasmid^48^ using T4 DNA ligase. The sgRNA target sequence (5′-ATTTATTTAGTAAAATGGAA-3′) is positioned at bps +70 to +89 within the *pfhp1* coding sequence and was identified using the CHOPCHOP web tool (http://chopchop.cbu.uib.no)^78,79^. The donor plasmid pFdon-N31 was generated by Gibson assembly joining four PCR fragments encoding (1) the pFdon plasmid backbone^48^ digested with *Sal*I and *EcoR*I; (2) the 103 bp fragment encoding the *sera2* intron:loxP fragment amplified from pD_SIP2xGFP plasmid (I. Niederwieser, unpublished) using primers F139 and R143; (3) a 5′ homology region (5′ HR) spanning bps −490 upstream of the start codon to +87 in the *pfhp1* coding sequence amplified from 3D7 gDNA using primers F147 and R145; and (4) a 3′ HR spanning bps +88 to +756 of the *pfhp1* coding sequence amplified from 3D7 gDNA using primers F146 and R144 (the *pfhp1* coding sequence is 798 bps long). For transfection, 50 µg of each plasmid (pHF-gC-guide31 and pFdon-N31) were mixed and co-electroporated into DiCre-expressing 3D7/1G5DC parasites^40^. Transfected parasites were selected with 4 nM WR99210 for six days and then cultured in absence of drug selection until transgenic populations were established.

In the second step, 3D7/N31DC parasites were transfected again to generate parasite lines 3D7/HP1-3M, 3D7/HP1-HIM and 3D7/HP1-Control that carry a second *sera2* intron:loxP sequence directly downstream of the endogenous *pfhp1* STOP codon, followed by a recodonised mutated (HP1-3M and HP1-HIM) or wild type (HP1-Control) *pfhp1-gfp* sequence using the following cloning steps. First, we constructed the pBF-gC-guide250 plasmid by inserting two annealed complementary oligonucleotides (F-g250 and R-g250) encoding the sgRNA target sequence at the 3′ end of the *pfhp1* coding sequence and appropriate single-stranded overhangs into the *Bsa*I-digested pBF-gC SpCas9 plasmid^48^ using T4 DNA ligase. The sgRNA target sequence (5′-AAAAAATTTAAGAGTTCCTG-3′) is positioned at bps +751 to +770 within the *pfhp1* coding sequence (negative strand) and was identified using CHOPCHOP (http://chopchop.cbu.uib.no). Second, we constructed the three donor plasmids. The pD-HP1-Control plasmid was constructed by Gibson assembly joining two PCR fragments. The first fragment was amplified from the plasmid pD-PfHP1-KO (see Supplementary Methods) using primers F162 and R143 and contains, in the following order, the *gpf* coding sequence ending with a STOP codon, a 3′ HR spanning the 824 bps directly downstream of the *pfhp1* STOP codon, the pD plasmid backbone^72^, a 5′ HR spanning bps +88 to +798 of the *pfhp1* coding sequence carrying eight synonymous mutations between bps +757 to +798 (see Supplementary Methods) and ending with a STOP codon followed by the 103 bp *sera2* intron:loxP element. The second PCR fragment was amplified from a plasmid containing a synthetic recodonized *pfhp1* sequence (pUC57-re-*pfhp1*) (GenScript™) (see Supplementary Fig. 3 and Supplementary Methods) using primers F164 and R165 and spans bps +88 to +798 of the *pfhp1* coding sequence omitting the STOP codon.

The pD-HP1–3M plasmid was constructed by Gibson assembly joining four PCR fragments encoding (1) the 5′ HR spanning bps +88 to +798 of the *pfhp1* coding sequence ending with a STOP codon followed by the 103 bp *sera2* intron:loxP element and bps +88 to +384 of the recodonised *pfhp1* sequence amplified from the pD-PfHP1-Control plasmid using primers F158 and R168, the latter of which introduces the S122/125A mutations into PfHP1; (2) a fragment spanning bps +367 to +798 of the recodonised *pfhp1* sequence amplified from pUC57-re-*pfhp1* (GenScript™) using primers F91 and R165, the former of which introduces the S125/129A mutations into PfHP1; (3) the *gfp* coding sequence ending with a STOP codon followed by the 3′ HR amplified from the pFdon-C-loxP-g250 vector (see Supplementary Methods) using primers F162 and R163; and (4) the pD plasmid backbone amplified from pUC19 using primers PCRA_F and PCRA_R^72^.

Finally, the pD-HP1-HIM plasmid was constructed in a two-step process. First, a fragment containing, in the following order, bps +88 to +798 of the recodonised *pfhp1* sequence encoding the S89/92/122/125/129/136/174A mutations followed by the *gfp* sequence ending with a stop codon and the 3′ HR was generated by Gibson assembly joining four fragments encoding (1) bps +88 to +280 of recodonised *pfhp1* amplified from pD-PfHP1-Control using primers F164 and R172, the latter of which introduces the S89/92A mutations; (2) bps +261 to +409 of the recodonised *pfhp1* sequence amplified from pD-PfHP1–3M (containing the S122/125/129A mutations) using primers F171 and R174, the former of which introduces the S89/92A mutations and the latter of which introduces the S136A mutation; (3) bps +390 to +535 of the recodonised *pfhp1* sequence amplified from pD-PfHP1-Control using primers F173 and R176, the former of which introduces the S136A mutation and the latter of which introduces the S174A mutation; and (4) a fragment containing, in the following order, bps +520 to +798 of the recodonised *pfhp1* sequence, the *gfp* coding sequence ending with a STOP codon and 3′ HR amplified from pD-PfHP1-Control using primers F175 and R163, the former of which introduces the S174A mutation. Second, the resulting fragment from the first step was used as template for a second round of PCR amplification using primers F164 and R163 and subjected to a second Gibson assembly joining with two other PCR fragments, namely the pD plasmid backbone amplified from pUC19 using primers PCRA_F and PCRA_R^72^, and a fragment containing the 5′ HR followed by the *sera2* intron:loxP amplified from the pD-PfHP1-Control using primers F158 and R143.

For each of the three transfections, 50 µg of the pBF-gC-guide250 plasmid was mixed with 50 µg of either pD-PfHP1-Control, pD-PfHP1–3M or pD-PfHP1-HIM and transfected by electroporation into the 3D7/N31DC parasite line as described above. Transfected parasites were selected with 5 µg/ml BSD-S-HCl for 10 days and then cultured in absence of drug pressure until transgenic populations were established. All oligonucleotide sequences used for the cloning of the CRISPR/Cas9 and donor plasmids are provided in Supplementary Table 3. The nucleotide sequence of recodonized *pfhp1* is provided in Supplementary Fig. 3.

### Nucleic acid isolation and diagnostic PCRs and reverse transcription PCRs

To confirm correct editing of the *pfhp1* locus we performed PCRs using the KAPA HiFi HotStart enzyme (Roche Sequencing Store) on gDNA isolated from the transgenic cell lines. To evaluate the excision efficiency after rapamycin treatment, diagnostic PCRs were performed on gDNA isolated 24–36 hours post rapamycin treatment^80^. To evaluate the splicing efficiency of the *sera2* intron:loxP from the *pfhp1* open reading frame, total RNA from the 3D7/N31DC mother cell line and the rapamycin-treated phosphomutant cell lines were isolated using Ribozol (Amresco) according to the manufacturer’s instruction. cDNA was then synthesized using oligo(dT) primers (RetroScript, Invitrogen) and PCRs on cDNA were performed using primers F106 and R107 that amplify the entire coding sequence. All transfection plasmids generated in this study have been validated by Sanger sequencing. All transfection plasmids have been designed and Sanger sequencing results analysed using the SnapGene software (from GSL Biotech; available at snapgene.com). All primer sequences used for PCR are listed in Supplementary Table 3.

### Induction of DiCre recombinase-mediated DNA excision by rapamycin treatment

Parasites were synchronized twice 16 hours apart to obtain an eight-hour growth window (16–24 hpi). After re-invasion parasites were synchronized again at 0–8 hpi (generation 1) and split into two equal populations, one of which was treated with 0.02% v/v of DMSO (negative control) and the other half was treated with 100 nM rapamycin for 1 hour^80^. The cultures were then spun down, washed with an equal volume of culture medium, resuspended in culture medium and returned to culture.

### SDS-PAGE and immunoblotting

After DMSO or rapamycin treatment in generation 1, parasites were allowed to complete schizogony and re-invasion. At 16–24 hpi in generation 2, parasites were released from iRBCs by 0.15% saponin/PBS complemented with 1X protease inhibitor (Roche Diagnostics). After washing 2–3 times in ice-cold PBS, parasite pellets were lysed in 70 °C pre-heated Urea/SDS buffer (8 M Urea, 5% SDS, 50 mM Bis-Tris, 2 mM EDTA, 25 mM HCl at pH 6.5 supplemented with 2 mM DTT and 1X protease inhibitor). Whole parasite protein lysates were separated on NuPage 4–12% Bis-Tris gels (Novex) and analyzed by Western blot using mouse mAb α-GFP (Roche Diagnostics #11814460001), 1:1000; rabbit α-PfHP1^31^, 1:5’000; rabbit α-Histone 4 (Abcam ab10158), 1:10’000. The blot shown in Fig. 3d was first probed with α-GFP antibodies, stripped in 2% SDS, 62.5 mM Tris-HCl (pH 6.8), 100 mM β-mercaptoethanol for 30 min at 60 °C, and re-probed with rabbit α-PfHP1 and α-Histone 4 antibodies simultaneously.

### Live cell fluorescence imaging

To quantify the efficiency of *pfhp1* excision after rapamycin treatment, live cell fluorescence microscopy was performed as described before^81^ with minor modifications using Hoechst (Merck) at a final concentration of 5 µg/ml to stain the nuclei. Excision efficiency was determined as the percentage of GFP-positive schizonts at 40–48 hpi in generation 1 (>100 schizonts counted per experiment). Images were taken at 63-fold magnification on a Leica DM 5000B microscope with a Leica DFC 300 FX camera, acquired via the Leica IM 1000 software, processed using ImageJ software (https://imagej.nih.gov/ij). For each experiment, images were acquired and processed with identical settings.

### Parasite multiplication assay

Parasites were tightly synchronized twice 16 hours apart, split into two equal populations after re-invasion at 0–8 hpi (generation 1), of which one half was treated with DMSO (negative control) and the other half was induced for DiCre recombinase-mediated DNA excision by rapamycin treatment as described above. Giemsa smears were prepared to determine the parasitaemia at 16–24 hpi (generation 1). Giemsa-stained smears were prepared every second day onwards for three generations. Parasitaemia was counted by visual inspection of Giemsa-stained blood smears (>3′000 RBCs counted per experiment). Multiplication rates were determined as the parasitaemia observed in the following generation divided by the parasitaemia observed in the previous generation.

### Gametocyte conversion assay

Parasites were tightly synchronized twice 16 hours apart and split into two equal populations after re-invasion at 0–8hpi (generation 1), of which one half was treated with DMSO (negative control) and the other half was induced for DiCre recombinase-mediated DNA excision by rapamycin treatment as described above. At 16–24 hpi in the subsequent generation (day 1 of gametocytogenesis), cultures were treated with 50 mM N-acetyl-D-glucosamine (GlcNAc) for six days to eliminate asexual parasites^58,59^ and then cultured with normal culture medium for another 4–6 days to observe gametocyte maturation. Gametocytaemia was determined on day 6 by visual inspection of Giemsa-stained blood smears. Sexual conversion rates were determined as the gametocytaemia observed on day 6 as a proportion of the total parasitaemia observed on day 1.

## Supplementary information

Supplementary Information

Dataset 1

Dataset 2

## Data Availability

All data generated or analysed during this study are included in this published article (and its Supplementary Information files).
