# Supplementary material for: Mapping and functional analysis of heterochromatin protein 1 phosphorylation in the malaria parasite Plasmodium falciparum
Source: Sci Rep. 2019 Nov 13;9:16720. doi: 10.1038/s41598-019-53325-9 (PMC6853920; doi:10.1038/s41598-019-53325-9)
Supplement: Supplementary file 1 — Supplementary Information [file 41598_2019_53325_MOESM1_ESM.pdf]

Supplementary Information

Mapping and functional analysis of heterochromatin protein 1 phosphorylation in the malaria parasite *Plasmodium falciparum*

Hai T. N. Bui[1,2], Igor Niederwieser[1,2], Megan J. Bird[3], Weiwen Dai[4,5], Nicolas M. B. Brancucci[1,2], Suzette Moes[6], Paul Jenoe[6], Isabelle S. Lucet[4,5], Christian Doerig[3,7], Till S. Voss[1,2,*]

[1]Department of Medical Parasitology and Infection Biology, Swiss Tropical and Public Health Institute, 4051 Basel, Switzerland

[2]University of Basel, 4003 Basel, Switzerland

[3]Department of Microbiology, Monash University, Clayton, Victoria 3800, Australia

[4]The Walter and Eliza Hall Institute of Medical Research, Parkville, Victoria 3052, Australia

[5]Department of Medical Biology, University of Melbourne, Parkville, Victoria, 3052, Australia

[6]Biozentrum, University of Basel, 4056 Basel, Switzerland

[7]Centre for Chronic Inflammatory and Infectious Diseases, School for Health and Biomedical Sciences, RMIT University, Bundoora Victoria 3083, Australia

[*]Corresponding author: till.voss@swisstph.ch

Supplementary Methods

Generation of the pFdon-C-loxP-g250 donor plasmid. The pFdon-C-loxP-g250 plasmid was constructed by Gibson assembly joining five DNA fragments. The first fragment represents the pFDon plasmid[1] digested with *Sal*I and *Ecor*I. The second fragment represents a *pfhp1* 5' HR spanning bps +88 to +798 omitting the stop codon and carrying eight synonymous mutations between bps +757 to +798 [(C→T)T(C→A)TTAAATTT(T→C)TTATTATCAAGA(C→T)TAAGATA(C→T)(C→A)G (T→A)ACAGC(T→G)], followed by 28 bps of the *sera2* intron:loxP element. This 5' HR was generated in a four-step PCR process. First, a PCR fragment containing six synonymous mutations within the region spanning bps +757 to + 790 of the *pfhp1* sequence (of which three are located within the sgRNA target sequence) was amplified from 3D7 gDNA using primers F133 and R138. This sequence was then used as template for a second round of amplification adding two more mutations within bps +792 to +798 of the *pfhp1* sequence followed by six bps of the *sera2* intron:loxP sequence (primers F133 and R138.1). The second sequence was then used as template for a third round of amplification adding nine more bps of the *sera2* intron:loxP sequence (primers F133 and R138.2). The third sequence was used as template for the final round of amplification to add in total 28 bps of the *sera2* intron:loxP sequence to serve as overhang for subsequent Gibson assembly (primers F133 and R138.3). The third fragment represents the *sera2* intron:loxP sequence followed by bps +1 to +356 of the *gpf* coding sequence amplified from the pD_SIP2xGFP plasmid (Igor Niederwieser, unpublished) using primers F139 and R148. The fourth fragment spans bps +336 to +714 of the *gfp* coding sequence ending with a stop codon and a Gibson assembly overhang amplified from the pD_SIP2xGFP plasmid (Igor Niederwieser, unpublished) using primers F149 and R136. The fifth and final fragment for Gibson assembly represents a *pfhp1* 3' HR spanning the 824 bps directly downstream of the stop codon amplified from 3D7 gDNA using primers F71 and R134. All oligonucleotide sequences used for the cloning of the pFdon-C-loxP-g250 plasmid are provided in Supplementary Table 3.

Generation of the pD-PfHP1_KO donor plasmid. The pD-PfHP1-KO donor plasmid was constructed by Gibson assembly joining three PCR fragments encoding (1) the plasmid backbone pD amplified from pUC19 using primers PCRA_F and PCRA_R[2], (2) a 5' HR spanning bps +88 to +798 of the *pfhp1* coding sequence ending with a stop codon carrying eight synonymous mutations within the region spanning bps +757 to +798 amplified from the pFdon-C-loxP-g250 plasmid (see above) using primers F158 and R159; and (3) a PCR product amplified from the pFdon-C-loxP-g250 plasmid using primers F139 and R163 and representing, in the following order, the 103 bp *sera2* intron:loxP element, the *gfp* coding sequence ending with a stop codon and a 3' HR sequence spanning 824 bps directly downstream of the *pfhp1* stop codon. All oligonucleotide sequences used for the cloning of the pD-PfHP1-KO plasmid are provided in Supplementary Table 3.

Supplementary Figures

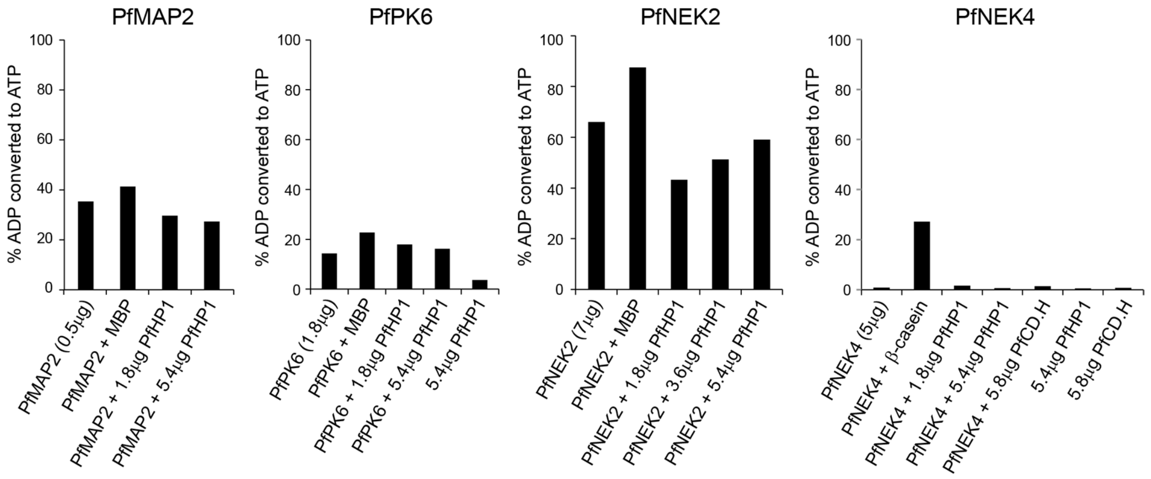

Supplementary Figure 1. ADP Glo *in vitro* kinase assay results. Based on ADP-Glo kinase assays PfMAP2, PfPK6, PfNEK2 and PfNEK4 do not specifically phosphorylate PfHP1 and PfCD.H *in vitro*. The percentage of ADP converted back into ATP (y-axis) is a surrogate measure for kinase activity (i.e. the relative amount of ATP consumed in the kinase reaction). MBP was included as a positive control substrate for PfMAP2, PfPK6 and PfNEK2, β-casein was included as a positive control substrate for PfNEK4. Recombinant PfHP1 and PfCD.H in absence of recombinant kinases were used as negative controls. All kinases except PfNEK4 show substantial autophosphorylation activity. Values represent the results of a single experiment each.

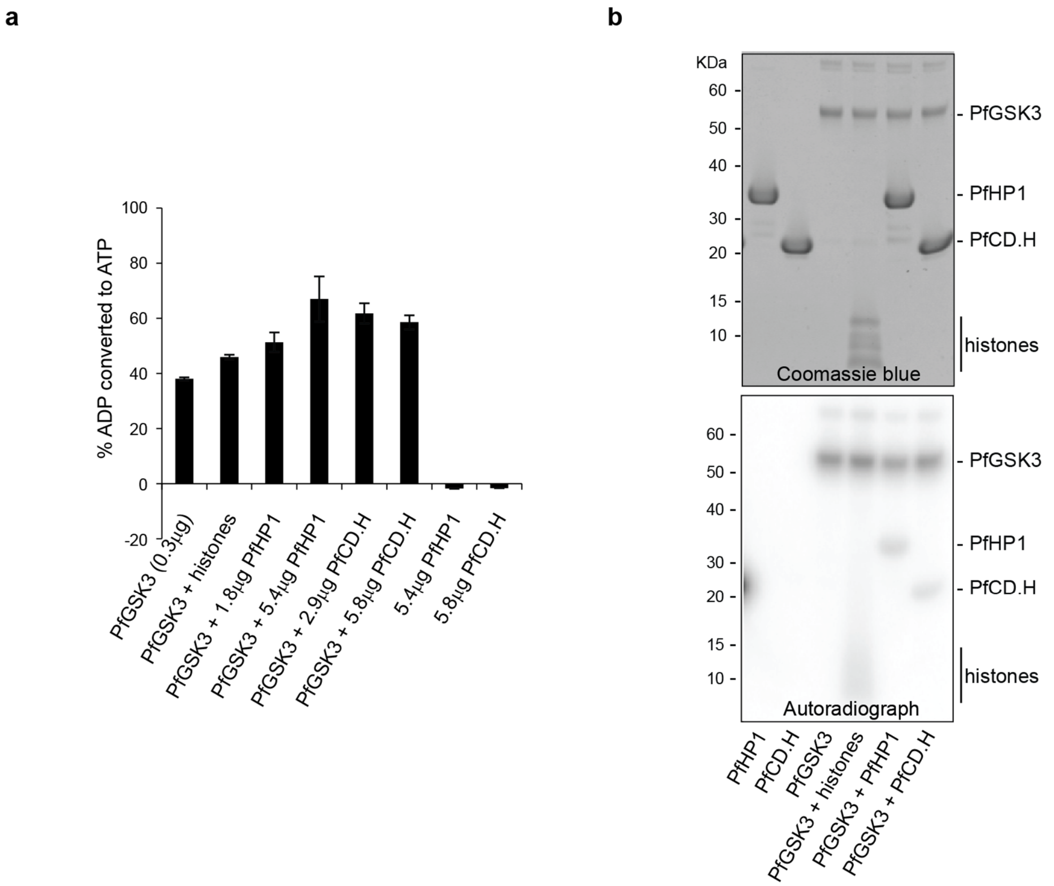

Supplementary Figure 2. PfGSK3 phosphorylates PfHP1 and PfCD.H *in vitro*. **(a)** ADP-Glo kinase assay. The percentage of ADP converted back into ATP (y-axis) is a surrogate measure for kinase activity (i.e. the relative amount of ATP consumed in the kinase reaction). Recombinant PfGSK3 showed strong autophosphorylation activity (column 1). Histones from calf thymus were used as positive control substrates (column 2). The addition of PfHP1 and PfCD.H increased the ATP consumption compared to addition of the control substrate (columns 3 to 6). Recombinant PfHP1 and PfCD.H in absence of PfGSK3 were used as negative controls (columns 7 and 8). The amount of PfGSK3 and substrates used in the assay is indicated. Values represent the results from two replicate reactions. Error bars represent SD. **(b)** γ-P^{32}-ATP kinase assay. Cropped sections of a Coomassie-stained gel (top) and the corresponding autoradiogram (bottom) of the *in vitro* γ-P^{32}-ATP PfGSK3 kinase assay performed with recombinant PfHP1 and PfCD.H substrates. Recombinant PfGSK3 showed strong autophosphorylation activity (lanes 3-6). Histones from calf thymus were used as positive control substrates (lane 4). PfGSK3 phosphorylated the control and the PfHP1 and PfCD.H test substrates (lanes 4-6). Recombinant PfHP1 and PfCD.H in absence of PfGSK3 were used as negative controls (lanes 1 and 2; the results for the negative control samples are the same as those shown in Fig. 2c). The full-size Coomassie-stained gel and autoradiogram are shown in Supplementary Fig. 6.

a

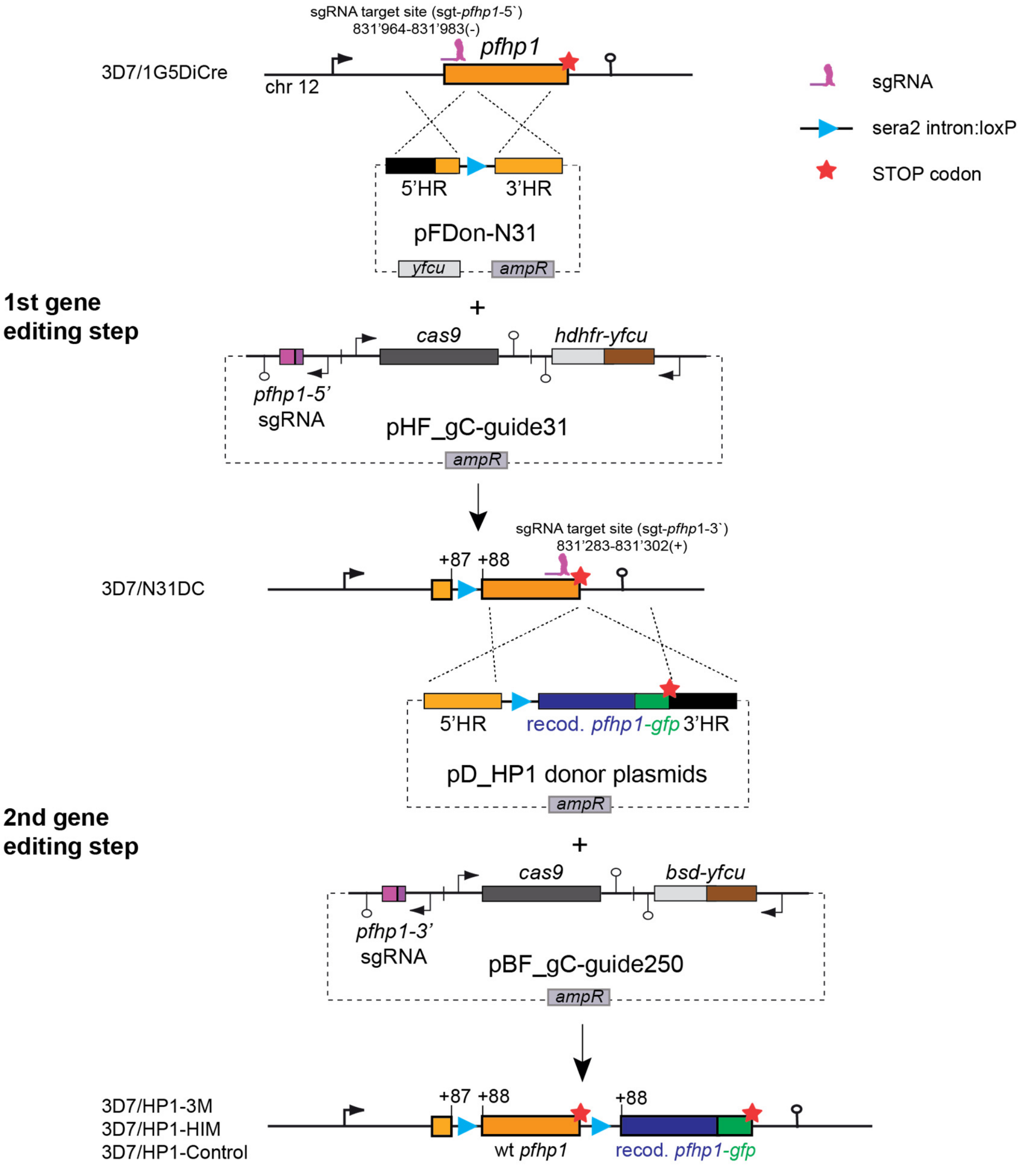

b

```
pfhp1     ATGACAGGGTCAGATGAAGAATTTGAAATTGGTGATATACTTGAAATAAAAAAAAAGAAGAATGGTTTTATTTATTTAGTAAAATGGAAAGGAT  94
           M  T  G  S  D  E  E  F  E  I  G  D  I  L  E  I  K  K  K  K  N  G  F  I  Y  L  V  K  W  K  G
pfhp1 re  ATGACAGGTAGTGATGAAGAATTTGAAATTGGAGATATATTAGAAATTAAAAAGAAGAAAAATGGTTTTATCTACTTAGTAAAATGGAAAGGAT  94
pfhp1     ATTCAGATGATGAGAATACTTGGGAACCCGAAAGTAATTTAATACATTTGACAACATTTAAGAAAAAGATGGAAAGCTTAAAAACGAATTTTTT 188
          Y  S  D  D  E  N  T  W  E  P  E  S  N  L  I  H  L  T  T  F  K  K  K  M  E  S  L  K  T  N  F  L
pfhp1 re  ATAGTGATGATGAAAATACATGGGAACCAGAATCAAATTTGATTCATTTGACAACTTTTAAAAAGAAAATGGAATCATTAAAAACAAATTTCTT 188
pfhp1     ATCGAAAGCTAATGAGACAAATGGTGATGGGAAAATTTTGAAAAATCATATATTAGCACCAACACAAGAAGACGATAGTATTAAATCAAAAGGT 282
            S  K  A  N  E  T  N  G  D  G  K  I  L  K  N  H  I  L  A  P  T  Q  E  D  D  S  I  K  S  K  G
pfhp1 re  ATCTAAAGCAAATGAAACAAATGGAGATGGTAAAATATTAAAGAATCATATTTTAGCTCCAACACAAGAAGATGATAGTATAAAATCAAAAGGT 282
pfhp1     AGAAGTTCCTTAGCACCCCGACGAAAAATGAGTAGAAAAAGTTTAACGAACAAACTAGAAAATAAAAAGAACTTATCTTTATCAGACAATTCTT 376
           R  S  S  L  A  P  R  R  K  M  S  R  K  S  L  T  N  K  L  E  N  K  K  N  L  S  L  S  D  N  S
pfhp1 re  AGAAGTTCATTAGCACCTAGAAGAAAAATGTCTAGAAAAAGTTTAACAAATAAGTTAGAAAATAAGAAAAATTTGTCATTATCTGATAATTCAT 376
pfhp1     TAAAAAAAAGTGATGAAGAAGATAATGAATCTGTAAAACATGAGAATCACGTTAATGATGGAAATTTATTAAATGTTGAAGATGTATATAGCGT 470
          I  K  K  S  D  E  E  D  N  E  S  V  K  H  E  N  H  V  N  D  G  N  L  L  N  V  E  D  V  Y  S  V
pfhp1 re  TAAAGAAAAGTGATGAAGAAGATAATGAATCTGTTAAACATGAAAATCATGTAAATGATGGAAATTTGTTAAATGTAGAAGATGTTTATAGTGT 470
pfhp1     TCGTATTAAAAATAAGAAATTGGAGTTTTTGGCTAGCTTGAAAAATGAATCTCCACAATGGGTAGAAGAAACAAATATTAGAAGAACTGGACAT 564
            R  I  K  N  K  K  L  E  F  L  A  S  L  K  N  E  S  P  Q  W  V  E  E  T  N  I  R  R  T  G  H
pfhp1 re  AAGAATTAAAAATAAGAAATTAGAATTTTTAGCTTCTTTAAAGAATGAAAGTCCACAATGGGTTGAAGAAACAAATATTAGAAGAACAGGTCAT 564
pfhp1     TTAAATATTAAAGTCAATGATTTTAAAAGATATGTAAGAAGAAAAAAAAGTTCTAGGGGTAATAGAATAGTTATCAAAAATCTACACAACGTTG 658
           L  N  I  K  V  N  D  F  K  R  Y  V  R  R  K  K  S  S  R  G  N  R  I  V  I  K  N  L  H  N  V
pfhp1 re  TTGAATATTAAAGTTAATGATTTTAAAAGATATGTTAGAAGAAAGAAAAGTAGTAGAGGAAATAGAATAGTAATTAAAAATTTGCATAATGTAG 658
pfhp1     GAGATGAATTATATATTTCGGTTATTCATAATATAAATAATAAAGAAATTCATAGTTTATATCCTTCCAAAGTTATTGAATATATTTATCCACA 752
          G  D  E  L  Y  I  S  V  I  H  N  I  N  N  K  E  I  H  S  L  Y  P  S  K  V  I  E  Y  I  Y  P  Q
pfhp1 re  GAGATGAATTATATATAAGTGTTATTCATAATATAAATAATAAGGAAATTCATAGTTTATATCCTTCAAAAGTAATAGAATATATATATCCTCA 752
pfhp1     GGAACTCTTAAATTTTTTATTATCAAGACTAAGATACCGTACAGCT                                                 798
            E  L  L  N  F  L  L  S  R  L  R  Y  R  T  A
pfhp1 re  AGAATTATTAAATTTCTTATTATCAAGATTAAGATATAGAACAGCA                                                 798
```

Supplementary Figure 3. Two-step CRISPR/Cas9-based gene editing strategy to generate DiCre-inducible PfHP1 phosphomutant cell lines. **(a)** First gene editing step: Schematic maps of the *pfhp1* locus (PF3D7_1220900) in 3D7/1G5DiCre parasites[3] (top), the co-transfected pFDon-N31 donor plasmid and pHF_gC-guide31 CRISPR/Cas9 transfection vector (center), and the modified *pfhp1* locus after CRISPR/Cas9-based gene editing in 3D7/N31DC parasites (bottom). The nucleotide positions of the sgt*_pfhp1-5'* sgRNA target sequence is indicated (chromosome 12 coordinates). The pFDon-N31 donor plasmid contains a 103 bp *sera2* intron:loxP element (light blue triangle)[4] flanked by two homology regions (HR) (orange, black) for homology-directed repair. The pHF_gC-guide31 plasmid contains expression cassettes for SpCas9 (dark grey), the sgRNA (purple) and the h*dhfr*-y*fcu* fusion selection marker (light grey-brown). Second gene editing step: Schematic maps of the *pfhp1* locus in 3D7/N31DC parasites (top), the co-transfected pD_HP1 donor plasmid (pD_HP1-Control is shown as an example) and pBF_gC-guide250 CRISPR/Cas9 transfection vector (center), and the modified *pfhp1* locus after CRISPR/Cas9-based gene editing in 3D7/HP1-3M, 3D7/HP1-HIM and 3D7/HP1-Control parasites (3D7/HP1-Control is shown as an example) (bottom). The nucleotide positions of the sgt*_pfhp1-3'* sgRNA target sequence is indicated (chromosome 12 coordinates). The pD_HP1 donor plasmids contain an assembly of the 103 bp *sera2* intron:loxP element (light blue triangle)[4] and the recodonised *pfhp1* sequence (dark blue) fused to *gfp* (green) flanked by two homology regions (HR) (orange, black) for homology-directed repair. The pBF_gC-guide250 plasmid contains expression cassettes for SpCas9 (dark grey), the sgRNA (purple) and the *bsd*-y*fcu* fusion selection marker (light grey-brown). Red stars represent STOP codons. Numbers refer to the nucleotide position within the *pfhp1* coding sequence. **(b)** Nucleotide sequences of the wild type (*pfhp1*) and recodonised (*pfhp1* re) *pfhp1* genes and amino acid sequence of PfHP1. Bases altered in the *pfhp1* re are highlighted in red letters. Numbers refer to the nucleotide position within the *pfhp1* coding sequence.

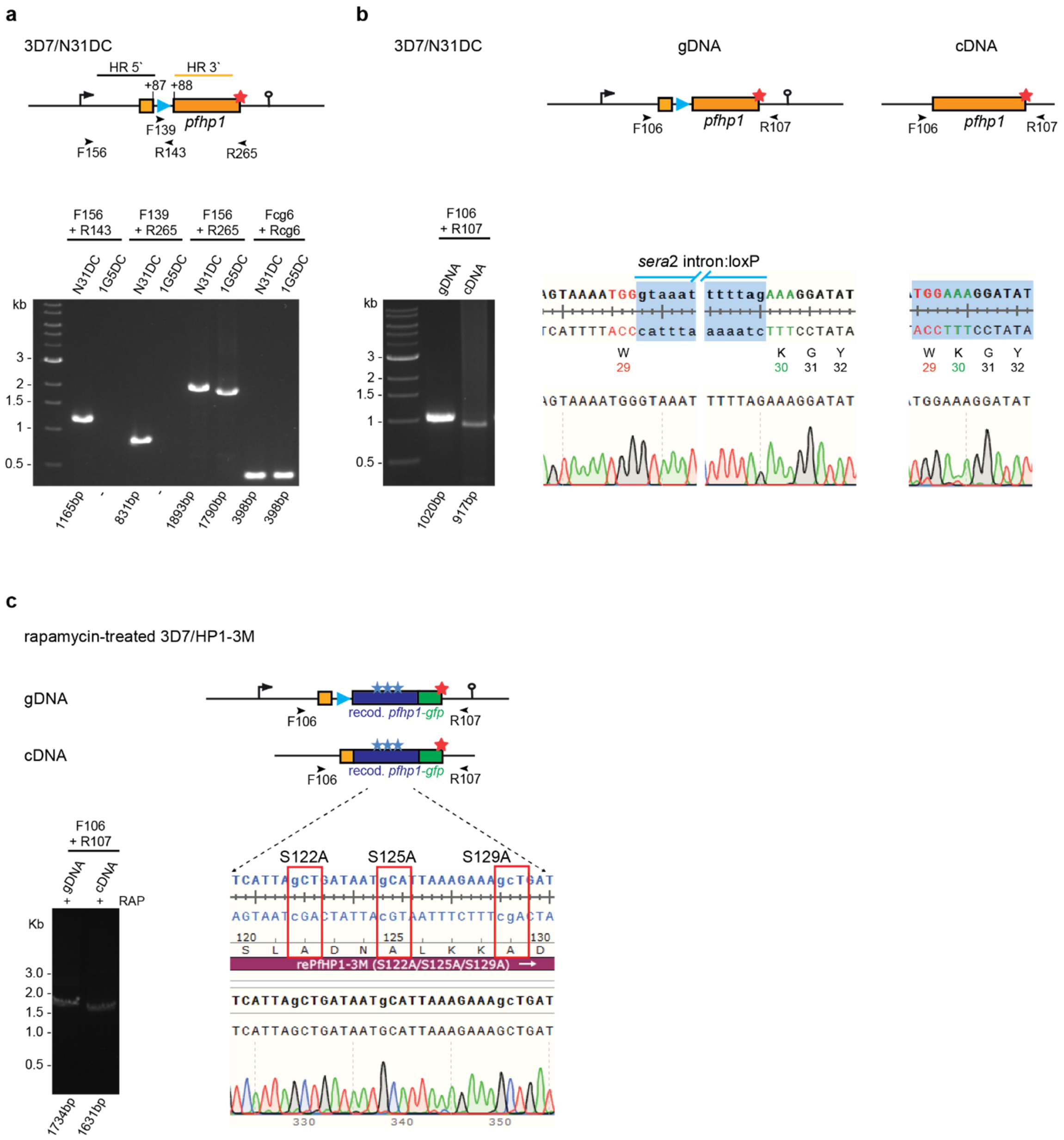

Supplementary Figure 4. Confirmation of successful gene editing by PCR and Sanger sequencing. **(a)** Top: Schematic of the CRISPR/Cas9-edited *pfhp1* locus in the 3D7/N31DC mother line carrying a 103 bp *sera2* intron:loxP element [4] inserted into the 5' end of the gene. The homology regions (HR) used for homology-directed repair of the Cas9-induced DNA double-strand break are indicated. The blue arrowhead indicates the position of *sera2* intron:loxP element. The red asterisk indicates the STOP codon. Numbers refer to nucleotide positions within the *pfhp1* coding sequence. The black arrowheads indicate the binding sites of the primers used to confirm correct gene editing of the *pfhp1* locus by PCR on gDNA. Bottom: PCR on gDNA isolated from 3D7/N31DC parasites and 3D7/1G5DiCre control parasites[3] confirms the correctly edited *pfhp1* locus in the 3D7/N31DC mother line. Primer pairs F156/R143 and F139/R265 deliver PCR products only from the 3D7/N31DC but not from the parental 3D7/1G5DiCre line (note that F139 and R143 bind specifically to the *sera2* intron:loxP element). Using

primers F156 and R265, the correctly edited *pfhp1* locus delivers a PCR product of 1893 bps in the 3D7/N31DC line and of 1790 bps in the parental 3D7/1G5DiCre line (i.e. prior to the insertion of the *sera 2* intron:loxP element). Primers targeting the *cg6* control locus (PF3D7_0709200) have been used as control. **(b)** Top: Schematic maps of the CRISPR/Cas9-edited *pfhp1* gene locus in the 3D7/N31DC mother line carrying a 103 bp *sera2* intron:loxP element (left) and the corresponding mRNA/cDNA sequence after splicing of the *sera2* intron:loxP element (right). The blue arrowhead indicates the position of *sera2* intron:loxP element. The red asterisk indicates the STOP codon. The black arrowheads indicate the binding sites of the F106 and R107 primers used to confirm splicing of the *sera2* intron:loxP element. Bottom left: PCR on gDNA and cDNA from 3D7/N31DC parasites confirms the correct splicing of the *sera2* intron:loxP element. The F106 and R107 primers bind up- and downstream of the *pfhp1* open reading frame and amplify a 1020 bp and 917 bp fragment from gDNA and cDNA, respectively. Bottom right: Sanger sequencing of PCR products amplified from gDNA and cDNA confirms the correct insertion of the *sera2* intron:loxP element into the *pfhp1* gene in 3D7/N31DC parasites and correct splicing of the *sera2* intron:loxP element, respectively. **(c)** Top: schematics of the CRISPR/Cas9-edited *pfhp1* locus in 3D7/HP1-3M parasites after rapamycin-induced DiCre-dependent replacement of endogenous wild type *pfhp1* with a recodonised mutated *pfhp1-3m-gfp* fusion gene and the corresponding mRNA/cDNA sequence after splicing of the *sera 2* intron:loxP element. The blue arrowhead indicates the position of *sera2* intron:loxP element. The red asterisk indicates the STOP codon. The blue asterisks indicate the relative position of mutations encoding serine-to-alanine substitutions in the 3D7/HP1-3M phosphomutant. Orange and blue boxes represent the wild type and recodonised *pfhp1* sequences, respectively. The black arrowheads indicate the binding sites of the F106 and R107 primers used to confirm splicing of the *sera2* intron:loxP element. Bottom left: PCR on gDNA and cDNA from rapamycin-treated 3D7/HP1-3M parasites confirms the correct splicing of the *sera2* intron:loxP element. The F106 and R107 primers bind up- and downstream of the *pfhp1-gfp* open reading frame and amplify a 1734 bp and 1631 bp fragment from gDNA and cDNA, respectively. Bottom right: Sanger sequencing of the RT-PCR product amplified from cDNA from rapamycin-treated 3D7/HP1-3M parasites confirms the successful introduction of the mutations encoding the S122A/S125A/S129A substitutions in the 3D7/HP1-3M phosphomutant.

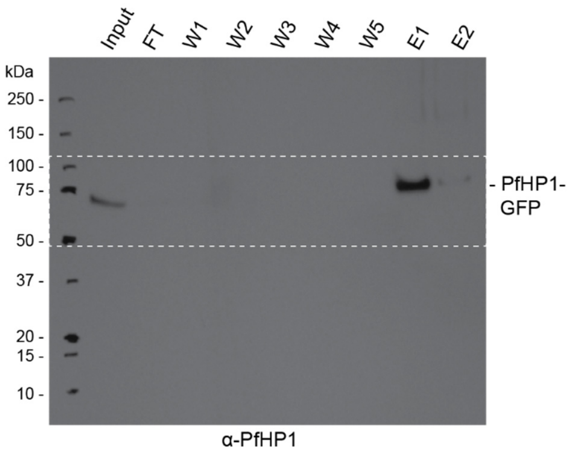

Supplementary Figure 5. Full size Western blot of the PfHP1-GFP IP samples probed with α-PfHP1 antibodies showing the presence of PfHP1-GFP in the input and elution samples. FT, flow through; W1-W5, wash 1-5; E1/E2, eluates 1 and 2. The dashed box represents the cropped section of the Western blot shown in Fig. 1a.

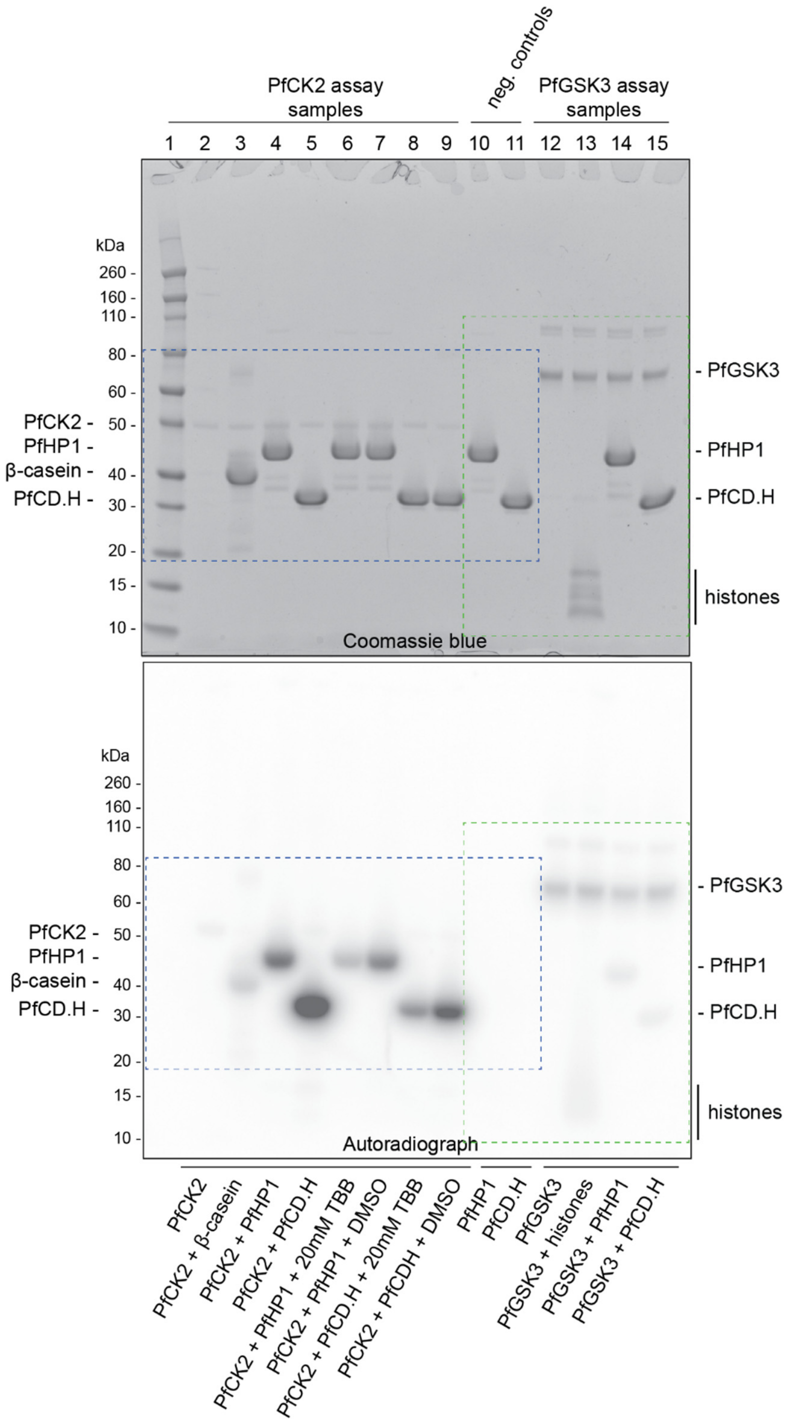

Supplementary Figure 6. γ-P32-ATP kinase assay. Full size Coomassie-stained SDS-PAGE gel (top) and the corresponding autoradiogram (bottom) of *in vitro* γ-P^{32}-ATP PfCK2 and PfGSK3 kinase assays performed with recombinant PfHP1 and PfCD.H substrates. β-casein was used as a positive control substrate for PfCK2 (lane 3). PfCK2 phosphorylated the control and the PfHP1 and PfCD.H test substrates (lanes 3-5). 20 μM TBB was used as a specific inhibitor of PfCK2[5,6] (lanes 6-9). Recombinant PfGSK3 showed strong autophosphorylation activity (lanes 12-15). Histones from calf thymus were used as positive control substrates for PfGSK3 (lane 13). PfGSK3 phosphorylated the control and the

PfHP1 and PfCD.H test substrates (lanes 13-15). Recombinant PfHP1 and PfCD.H in absence of PfCK2 and PfGSK3 were used as negative controls (lanes 10-11). The blue dashed boxes represent the cropped sections of the PfCK2 kinase assays results shown in Fig. 2c, the green dashed boxes represent the cropped sections of the PfGSK3 kinase assays results shown in Supplementary Fig. 2.

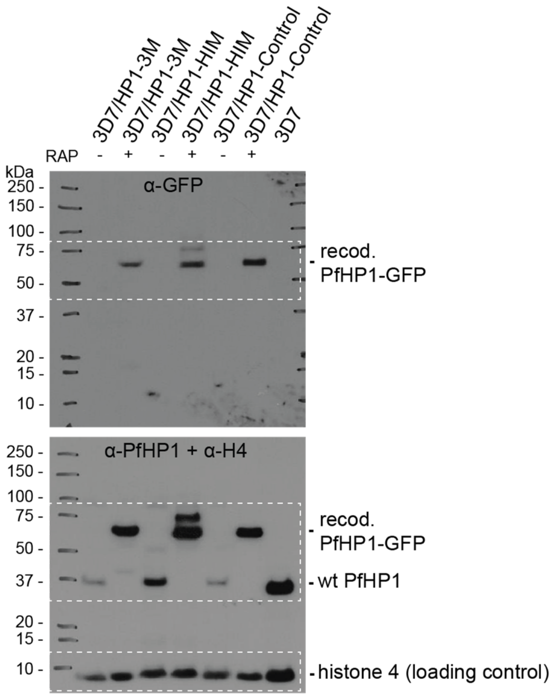

Supplementary Figure 7. Full size Western blots showing the expression of endogenous untagged PfHP1 and recodonised PfHP1-GFP in the progeny of DMSO- and rapamycin-treated 3D7/HP1-3M, 3D7/HP1-HIM and 3D7/HP1-Control parasites (16-24 hpi, generation 2) (64 hrs after rapamycin treatment). A sample harvested from 3D7 wild type parasites was used as negative control sample. α-histone 4 antibodies were used as loading control. RAP, rapamycin. The membrane was first probed with α-GFP antibodies (upper panel), stripped with 2% SDS, 62.5 mM Tris-HCl (pH 6.8), 100mM β-mercaptoethanol, and re-probed with rabbit α-PfHP1 and α-histone 4 antibodies simultaneously (lower panel). The dashed boxes represent the cropped sections of the Western blots shown in Fig. 3d.

Supplementary Tables

Supplementary Table 1. Mass spectrometry results of the PfHP1-GFP immunoprecipitation experiments. Proteins and peptides identified by Mascot and Sequest HT searches of PfHP1-GFP IPs from four independent IP experiments (#1-4) analysed by LC-MS/MS. Worksheets 2-5 list the proteins and peptides identified in the four IP samples by searching against a merged *P. falciparum*/human annotated protein database ("PfHs-merged"). Worksheets 6 and 7 list all PfHP1-derived peptides identified in IP samples 1 and 2 by searching the data against the PfHP1-GFP sequence ("HP1-GFP") only. The PfHP1 protein and PfHP1-derived peptides are highlighted in yellow and green, respectively. The table in worksheet 1 summarises all phosphopeptides identified in the individual searches with IP replicate samples 1, 2, 3 and 4 highlighted in green, red, blue and yellow, respectively. Known phosphorylation site motifs corresponding to the phosphorylated sites identified in PfHP1, and the kinases responsible for phosphorylating these motifs in other eukaryotes are indicated in columns E and F, respectively (http://www.hprd.org/PhosphoMotif_finder)[7].

Supplementary Table 2. Mass spectrometry results of the PfCK2 and PfGSK3 ADP-Glo *in vitro* kinase assays. Peptides and phosphopeptides identified by Mascot and Sequest HT searches of the ADP-Glo *in vitro* kinase assays with recombinant PfHP1 and PfCD.H phosphorylated with recombinant PfCK2 (worksheets 1-4), recombinant PfHP1 alone as negative control (worksheet 5) and recombinant PfGSK3 (worksheet 6). For the PfCK2-PfHP1 and PfCK2-PfCD.H assays, LC-MS/MS analysis has been performed on two replicate samples each [PfHP1-CK2 repl 1 and repl 2 (worksheets 1 and 2) and PfCD.H-CK2 repl 1 and repl 2 (worksheets 3 and 4)]. Phosphopeptides highlighted in pink, yellow and blue carry phosphorylated S122, S125 and S129, respectively.

Supplementary Table 3. List of all oligonucleotides used in this study.

Application	Oligo name	Sequence (5'-3')
Annealing	F-g31	TATTATTTATTTAGTAAAATGGAA
	R-g31	AAACTTCCATTTTACTAAATAAAT
	F-g250	TATTCATAATAAAATTAAGCTGTA
	R-g250	AAACCAGGAACTCTTAAATTTTTT
PCR gDNA/cDNA	F106	CCTACTTGTACACAAAATAAAAG
	R107	CTTATATAAATAAATTTACTACAG
	F119	GTGTGTGTTTAAGAAAAAATATG
	F156	GCAATAAGAAAAAAAATGGGAGG
	R157	CATGTAGCCAAAATATGTG
	R265	TATTCATAATAAAATTAAGCTGTA
	F-cg6	GTTCATGCTCCTCAACAAAG
	R-cg6	GAACAAATACATAAGAGCGC
PCR cloning transfection vectors	F71	TTTTATTATGCAAATATACATATATAC
	F91	GATAATGCATTAAAGAAAGCTGATGAAGAAGATAATGAATCTG
	F133	CAGTGAGCGAGGAAGCTTGTCGACAAAGGATATTCAGATGATGAG
	R134	CTTTTCTCTTGTGGATCCGAATTCGAGGTTAAAATTCTAAACTATATG
	R136	GTATATATGTATATTTGCATAATAAAATTATTTGTATAGTTCATCCATG
	R138	TATATCTTAATCTTGATAATAAGAAATTTAATAATTCCTGTGGATAAATATATTC
	R138.1	ATTTACCGCTGTTCTATATCTTAATCTTGATAATAAG
	R138.2	ATTTTTTTTATTTACCGCTGTTCTATATCTTAATC
	R138.3	GTTATTGTATATTATTTTTTTTATTTACCGCTGTTC
	F139	GTAAATAAAAAAAATAATATACAATAAC
	R143	CTAAAAGAATATAAAATATATAAATAT
	R144	CTTTTCTCTTGTGGATCCGAATTCTTCCTGTGGATAAATATATTC
	R145	GTTATTGTATATTATTTTTTTTATTTACCCATTTTACTAAATAAATAAAAC
	F146	ATATTTATATATTTTATATTCTTTTAGAAAGGATATTCAGATGATGAG
	F147	CAGTGAGCGAGGAAGCTTGTCGACACACCCCCAAAAGGCCGA
	R148	AGGGTATCACCTTCAAACTTGACTTCAGCACGTGTCTTGTAG
	F149	CAAGTTTGAAGGTGATACCCT
	F158	CGTTGGCCGATTCATTAATGAAAGGATATTCAGATGATGAG
	R159	GTTATTGTATATTATTTTTTTTATTTACTTACGCTGTTCTATATCTTAATC
	F162	ATGAGTAAAGGAGAAGAAC
	R163	CCTCTTCGCTATTACGCCAGGAGGTTAAAATTCTAAACTATATG
	F164	ATATTTATATATTTTATATTCTTTTAGAAAGGATATAGTGATGATGA
	R165	GTTCTTCTCCTTTACTCATTGCTGTTCTATATCTTAATC
	R168	TTTCTTTAATGCATTATCAGCTAATGACAAATTTTTCTTATTTTC
	F171	TGATGCAATAAAAGCTAAAGGTAGAAGTTCATTAG
	R172	CTTTAGCTTTTATTGCATCATCTTCTTGTGTTGGAG
	F173	TGAAGAAGATAATGAAGCAGTTAAACATGAAAATCATGTAAATG
	R174	CTGCTTCATTATCTTCTTCATCAG
	F175	GCACCACAATGGGTTGAAGAAAC
	R176	CAACCCATTGTGGTGCTTCATTCTTTAAAGAAGCTAAAAATTC
	PCRA_F	CTGGCGTAATAGCGAAGAGG
	PCRA_R	CATTAATGAATCGGCCAACG
PCR cloning expression vectors	Bsa_His_f	AAGGTCTCGGATCTCATCATCATCATCATCACGGG
	T7term	TGCTAGTTATTGCTCAGCGG
	HP1_F	ATGACAGGCTCAGATGAAGAATTTGAAATTGG
	HP1_Xho_R	ATATTTGCATTCTCGAGTTAAGCTGTACGG
	CDH_Xho_R	GTTCCTCGAGTTTATGTTTCTTCTACCCATTGTGG

References

1. Filarsky, M. *et al.* GDV1 induces sexual commitment of malaria parasites by antagonizing HP1-dependent gene silencing. *Science* **359**, 1259-1263 (2018).
2. Brancucci, N. M. B. *et al.* Lysophosphatidylcholine Regulates Sexual Stage Differentiation in the Human Malaria Parasite Plasmodium falciparum. *Cell* **171**, 1532-1544 e1515 (2017).
3. Collins, C. R. *et al.* Robust inducible Cre recombinase activity in the human malaria parasite Plasmodium falciparum enables efficient gene deletion within a single asexual erythrocytic growth cycle. *Mol. Microbiol* **88**, 687-701 (2013).
4. Jones, M. L. *et al.* A versatile strategy for rapid conditional genome engineering using loxP sites in a small synthetic intron in Plasmodium falciparum. *Sci. Rep* **6**, 21800 (2016).
5. Sarno, S. *et al.* Selectivity of 4,5,6,7-tetrabromobenzotriazole, an ATP site-directed inhibitor of protein kinase CK2 ('casein kinase-2'). *FEBS Lett* **496**, 44-48 (2001).
6. Holland, Z., Prudent, R., Reiser, J. B., Cochet, C. & Doerig, C. Functional analysis of protein kinase CK2 of the human malaria parasite Plasmodium falciparum. *Eukaryot. Cell* **8**, 388-397 (2009).
7. Amanchy, R. *et al.* A curated compendium of phosphorylation motifs. *Nat Biotechnol* **25**, 285-286 (2007).
